# Supplementary figures and images for: Mitochondrial fusion regulates proliferation and differentiation in the type II neuroblast lineage in Drosophila
Source: PLoS Genet. 2022 Feb 14;18(2):e1010055. doi: 10.1371/journal.pgen.1010055 (PMC8880953; doi:10.1371/journal.pgen.1010055)

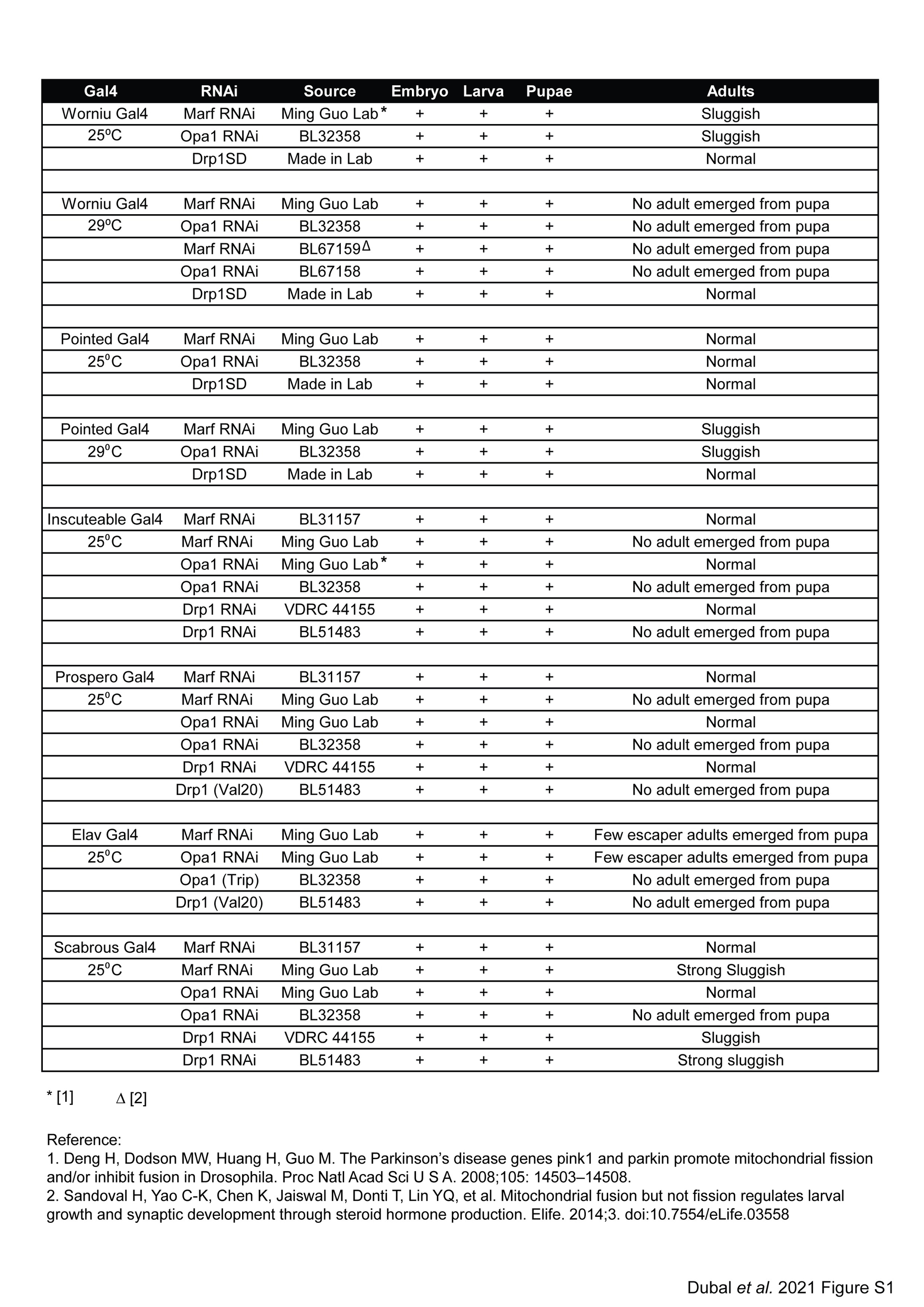

Supplement: S1 Fig — Various Gal4 drivers were crossed with RNAi depletion lines and a Drp1 dominant negative mutant of mitochondrial morphology genes at 25 or 29°C and lethality or behavioral phenotype was recorded in the adult. worniu-Gal4 (wor-Gal4), inscuteable-Gal4, prospero-Gal4 and scabrous-Gal4 expresses the Gal4 in all NBs, pnt-Gal4 expresses in type II NBs and elav-Gal4 expresses in neurons. Adult flies from crosses with wor-Gal4 (25°C) and pnt-Gal4 (29°C) with opa1 RNAi and marf RNAi were sluggish and the numbers obtained were at the expected frequency, no lethality was seen at the pupal stage. elav-Gal4 crosses gave lethality and few adults emerged. The opa1 RNAi BL32358 and opa1 RNAi2 BL67158 gave stronger phenotypes as compared to opa1 RNAi Ming Guo lab with inscuteable-Gal4, prospero-Gal4, elav-Gal4 and scabrous-Gal4. The marf RNAi Ming Guo lab and marf RNAi2 BL67159 gave stronger phenotypes as compared to marf RNAi BL31157 with inscuteable-gal4, prospero-Gal4 and elav-Gal4. We chose opa1 RNAi BL32358, opa1 RNAi2 BL67158, marf RNAi Ming Guo lab and marf RNAi BL67159 for further analysis. The Drp1 RNAi from VDRC did not show phenotypes and the Drp1 (BL51483) gave inconsistent results, hence we used a lab generated construct overexpressing the GTPase domain mutant Drp1SD for further analysis. (TIF) [file pgen.1010055.s001.tif]

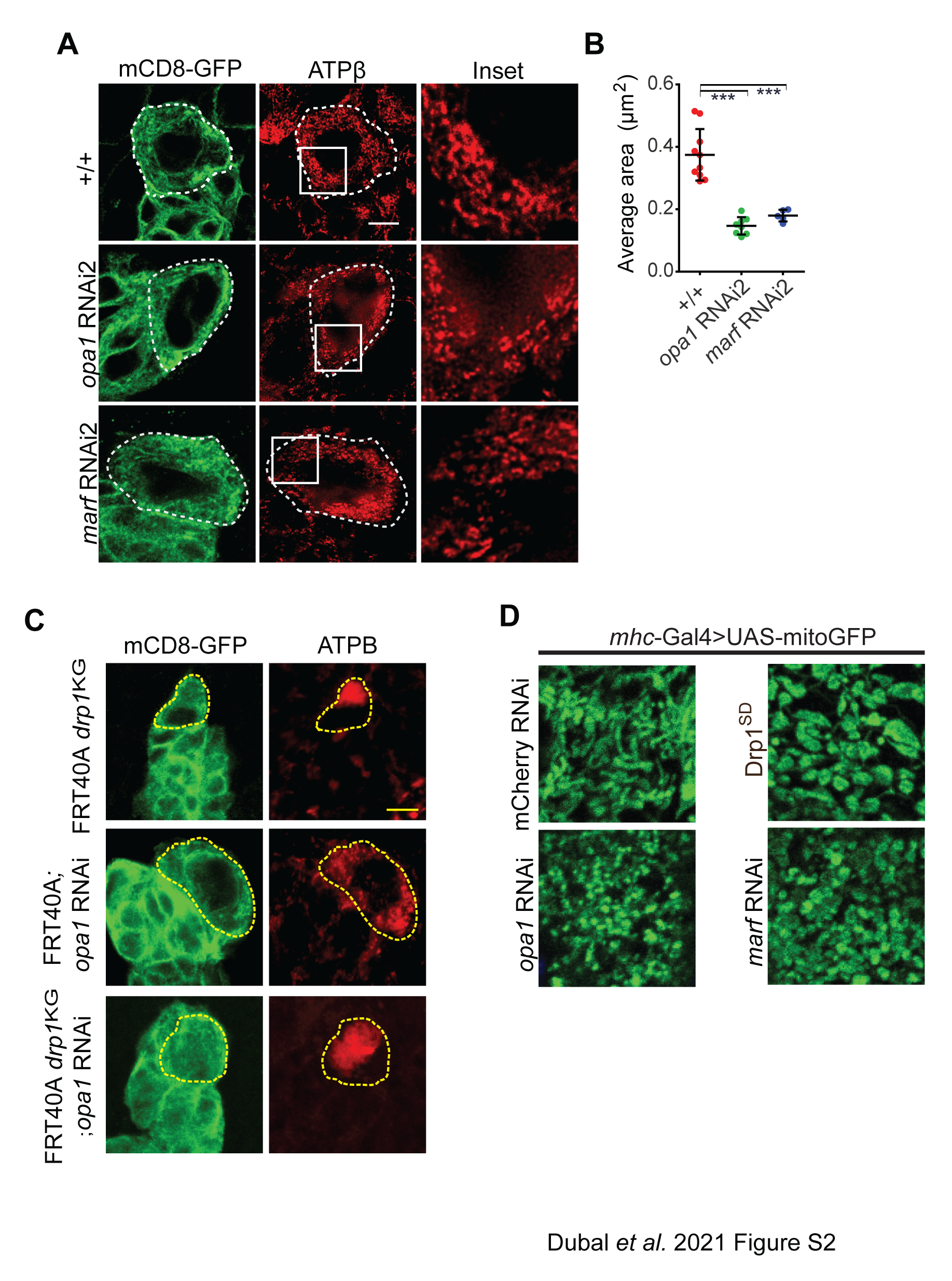

Supplement: S2 Fig — A, B:Mitochondrial morphology and distribution in type II NBs (white dotted line, magnified area shown in the panel on the right) stained with ATPβ (red) antibody using STED super resolution microscopy is shown in representative images with zoomed inset in the right panel (A). Control (100% tubular, 75 NBs, 22 brains), opa1 RNAi2 (100% fragmented, 14,4), marf RNAi2 (100% fragmented, 14,4). Average mitochondrial area quantification from type II NBs (B) in control (10 type II NBs, 4 brains), opa1 RNAi2 (8,4), marf RNAi2 (5,3). Scale bar- 5μm. C: Clonal analysis of NB clones (green) expressing drp1KG allele show clustered mitochondria stained with ATPβ antibody (red). FRT40A drp1KG (8 NB clones), FRT40A;opa1 RNAi (13), FRT40A drp1KG;opa1 RNAi (13). D: Representative confocal images of mitochondria labeled with mito-GFP (green) in larval muscles. Expression of opa1 and marf RNAi by mhc-Gal4 shows smaller mitochondria while Drp1SD shows large mitochondria compared to control. Representative images from a minimum of 3 larvae are shown, the phenotype of smaller mitochondria is at 100% in muscle 6 and 7 of segment A2 and A3 in opa1 and marf RNAi and of larger mitochondria in Drp1SD overexpression. Scale bar-3μm. (TIF) [file pgen.1010055.s002.tif]

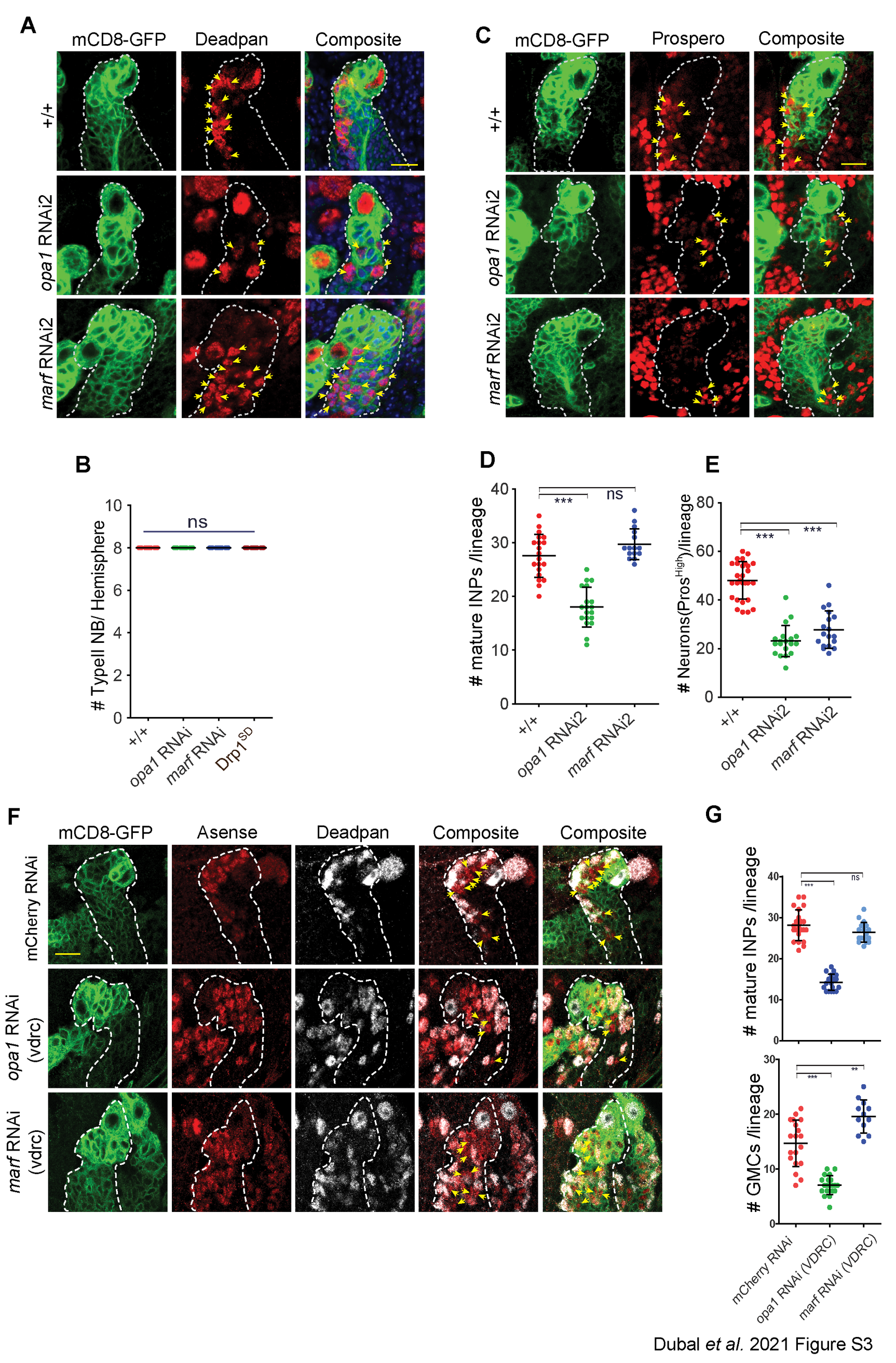

Supplement: S3 Fig — A,C:Type II NB lineages (yellow dotted line) showing expression of mCD8-GFP (green) and Dpn (red, yellow arrows) (A). Quantification of Dpn positive mature INPs (C) in control (20 NB lineages, 5 brains), opa1 RNAi2 (19,4), marf RNAi2 (15,4). Scale bar- 10μm. B: Quantification of number of type II NBs in control, opa1 RNAi, marf RNAi, Drp1SD (n = 15 brains each). C,E:Type II lineages (pnt-Gal4, mCD8-GFP, green) showing Pros positive INPs (red, yellow arrows) (C, yellow arrows). Quantification of Pros positive young neurons (E) in control (28 NB lineages,8 brains), opa1 RNAi2 (19,4), marf RNAi2 (18,4). Scale bar- 10μm. F-G: Representative type II NB lineages showing reduced numbers of mature INPs and GMCs in opa1 RNAi from VDRC stock center. Quantification of mature INPs (G) in mCherry RNAi, opa1 RNAi (VDRC), marf RNAi (VDRC). Analysis of numbers of GMCs (Dpn- Ase+, yellow arrows) in type II NB lineage in mCherry RNAi (19 type II NB lineages, 6 Brains), opa1 RNAi (VDRC) (20,5), marf RNAi (VDRC) (12,5). B,D,E& G: Graphs show mean ± sd. Statistical analysis is done using an unpaired t-test. ns- non significant, ***- p<0.001. (TIF) [file pgen.1010055.s003.tif]

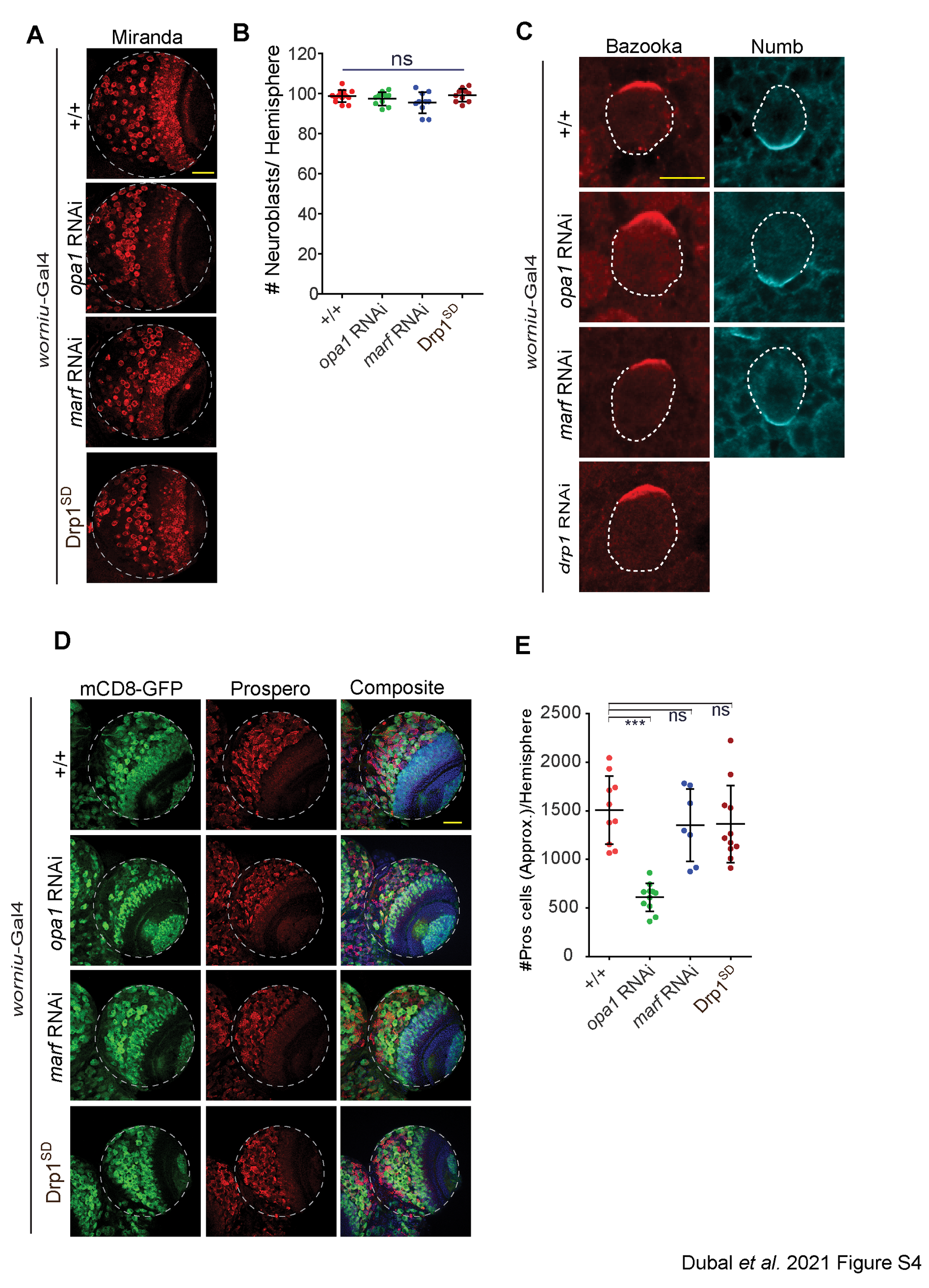

Supplement: S4 Fig — A, B: Representative confocal images of larval brain lobes stained for Miranda (red) show no change in NB number (A). Expression of opa1 RNAi, marf RNAi and Drp1SD was done by wor-Gal4. Quantification of NB number in larval brain lobes (B) of control (13 lobes,13 brains), opa1 RNAi (10,10), marf RNAi (10,10), Drp1SD (10,10). Scale bar- 50μm. C: Representative images showing apical Bazooka and basal Numb localization in control and mitochondrial dynamics mutants. Bazooka is present at the apical side (away from progenies) and Numb at the basal side (near the progenies) in dividing NBs of all genotypes. Scale bar- 10μm. D, E: Representative images of brain lobes showing reduced Pros positive cells in opa1 RNAi expressed with wor-Gal4, mCD8-GFP (D). Analysis of bright Pros positive cells (E) in control (10 lobes,10 brains), opa1 RNAi (11,11), marf RNAi (7,7), Drp1SD (11,11). Scale bar- 50μm. B, E: Graphs show mean ± sd. Statistical analysis was done by using unpaired t-test. ns- non significant, ***- p<0.001. (TIF) [file pgen.1010055.s004.tif]

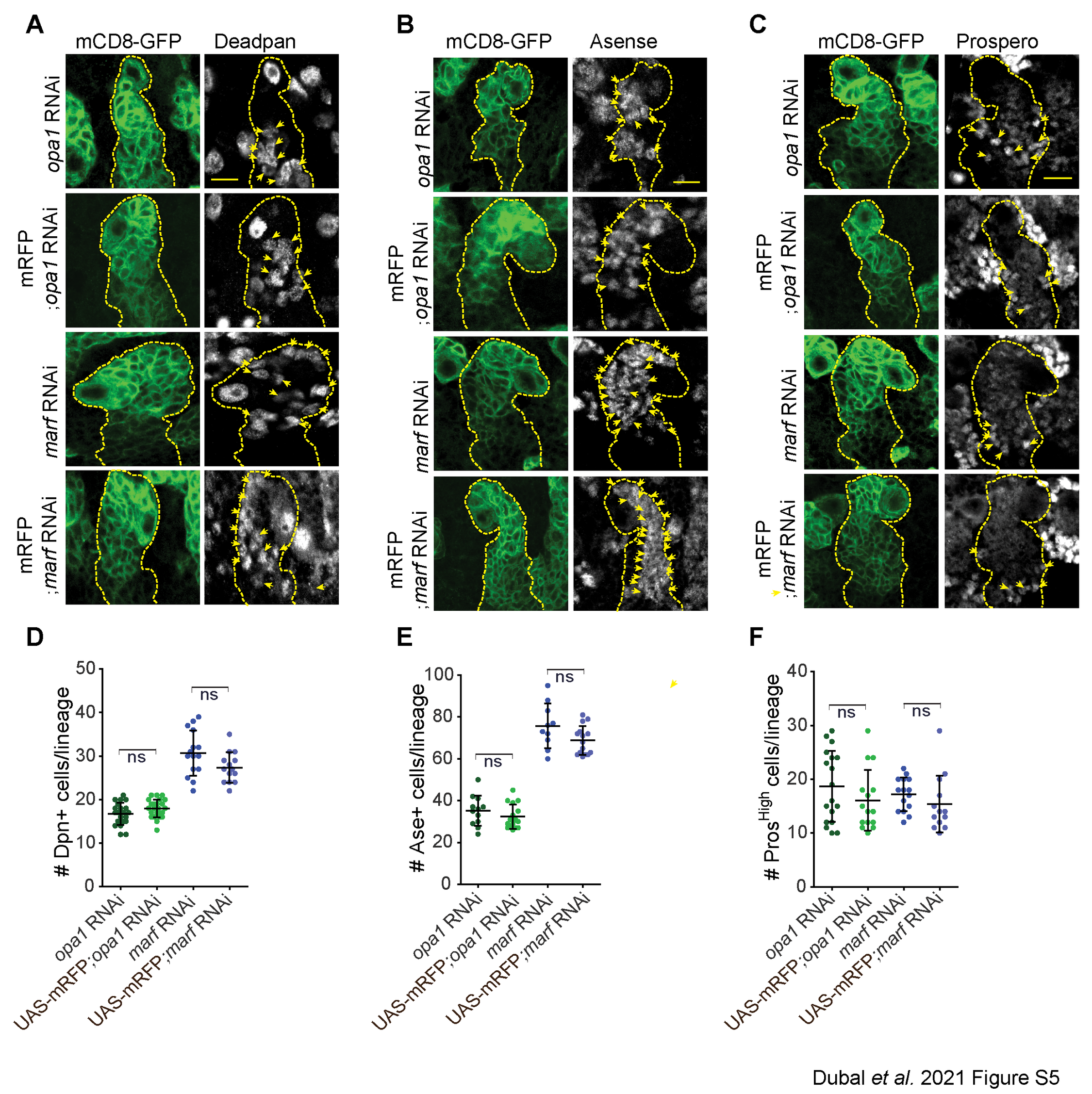

Supplement: S5 Fig — A, D: Representative confocal images of type II NB lineages showing expression of Dpn (grey scale) (A) in opa1 RNAi, mRFP;opa1 RNAi, marf RNAi, mRFP;marf RNAi. Quantification of Dpn positive cells (yellow arrows) (D) in opa1 RNAi (21 Type II NB lineages, 6 Brains), mRFP;opa1 RNAi (25,4), marf RNAi (15,8), mRFP;marf RNAi (15,3). Scale bar- 10μm. B, E: Representative confocal images of type II NB lineages showing Ase positive cells (grey scale) (B) in opa1 RNAi, mRFP;opa1 RNAi, marf RNAi, mRFP;marf RNAi. Quantification of Ase positive cells (yellow arrows) (E) in opa1 RNAi (12 Type II NB lineages, 5 Brains), mRFP;opa1 RNAi (16,3), marf RNAi (10,4), mRFP;marf RNAi (15,3). Scale bar- 10μm. C, F: Representative confocal images of type II NB lineages showing high levels of Pros expressing young neurons (grey scale) (C) in opa1 RNAi, mRFP;opa1 RNAi, marf RNAi, mRFP;marf RNAi. Quantification of brightly stained Pros positive young neurons (yellow arrows) (F) in opa1 RNAi (18 Type II NB lineages, 5 Brains), mRFP;opa1 RNAi (15,3), marf RNAi (15,5), mRFP;marf RNAi (13,3). Scale bar- 10μm. D-F: Graphs show mean ± sd. Statistical analysis was done by using unpaired t-test. ns- non significant. Values for opa1 RNAi and marf RNAi are repeated from Fig 2 for comparison. (TIF) [file pgen.1010055.s005.tif]

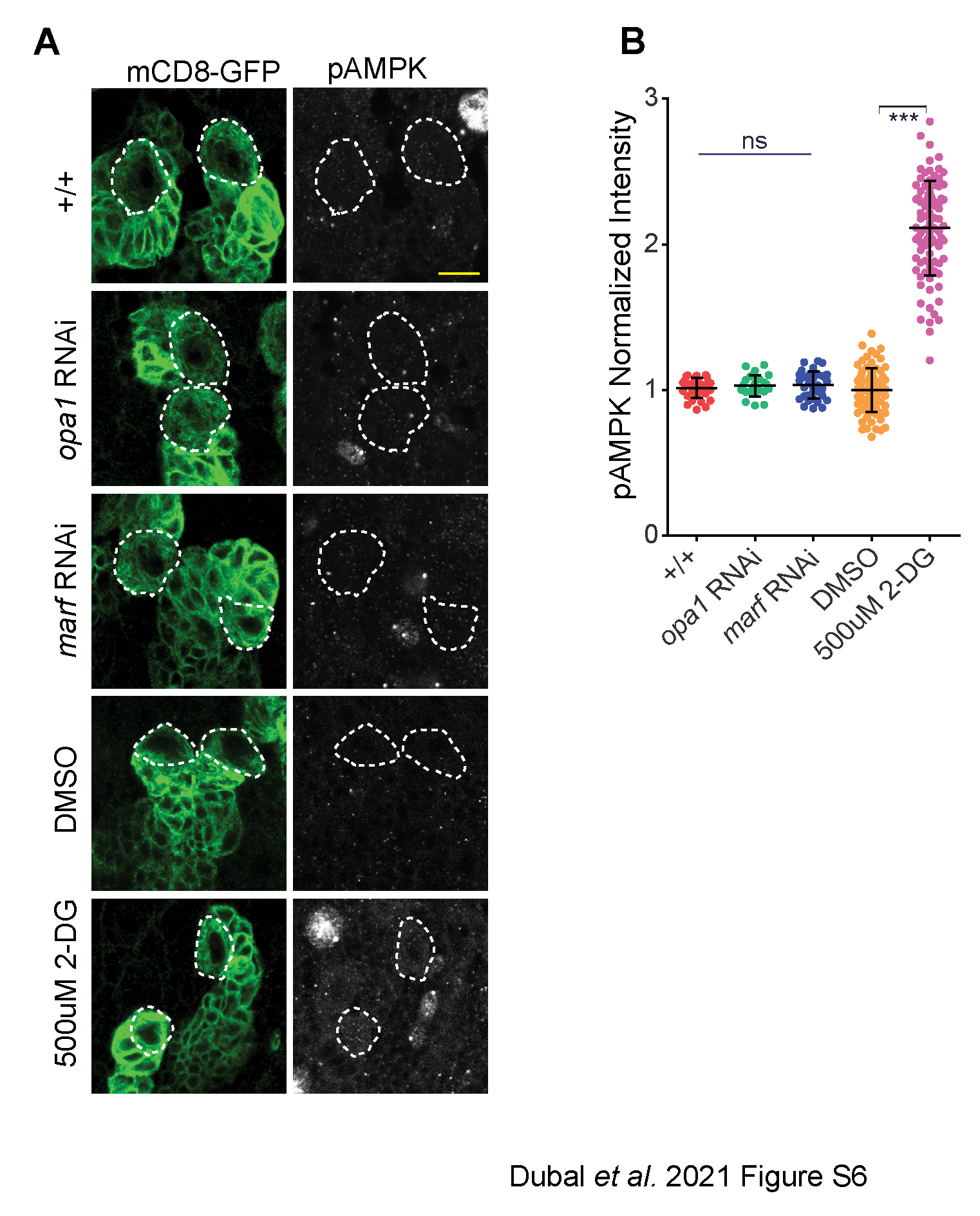

Supplement: S6 Fig — A, B: Representative images of type II NBs expressing opa1 and marf RNAi along with pnt-Gal4, mCD8-GFP did not show change in levels of pAMPK (pAMPK fluorescence is shown as a grey scale) (A). Brains of different genotypes were treated with 2-DG and were imaged for pAMPK fluorescence at the same time under the same imaging conditions. Type II NBs are marked by white dotted lines while lineages are marked by expression of mCD8-GFP (green). Analysis of pAMPK intensity normalised with neighboring control cells (B). +/+ (32 NBs,14 brains), opa1 RNAi (23,8), marf RNAi (33,8), DMSO (76,8), 2-DG (91,9). Scale bar- 10μm. (TIF) [file pgen.1010055.s006.tif]

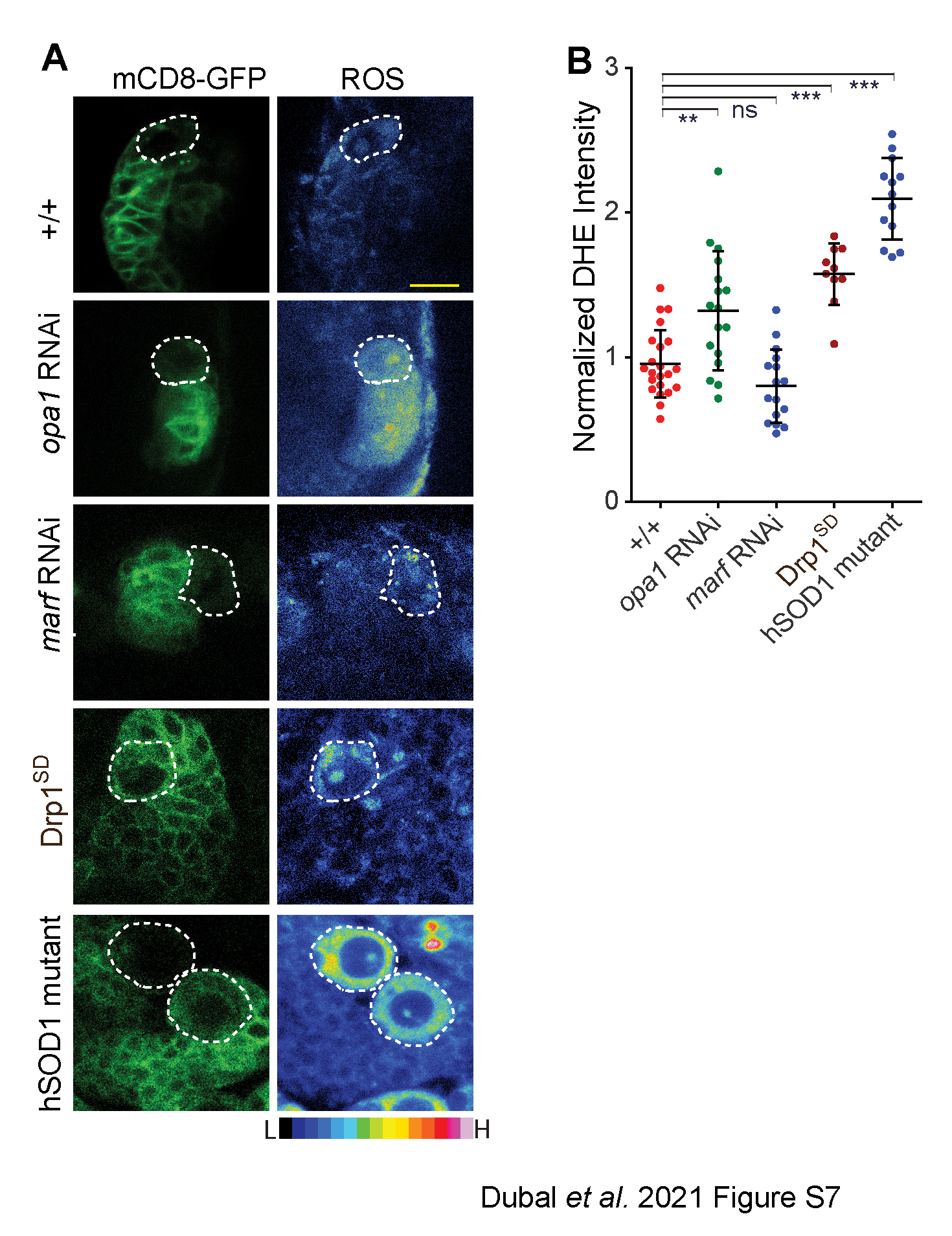

Supplement: S7 Fig — A, B: Representative images showing increased levels of ROS (rainbow scale) in type II NB (position marked by white dotted line) using pnt-Gal4, mCD8-GFP with different genotypes (A). Analysis of relative DHE fluorescence as a ratio to neighboring cells (B) in type II NBs, +/+ (22 NBs,6 Brains), opa1 RNAi (17,8), marf RNAi (17,8), Drp1SD (9,5), hSOD1 mutant overexpression (13,3). Scale bar- 10μm. (TIF) [file pgen.1010055.s007.tif]

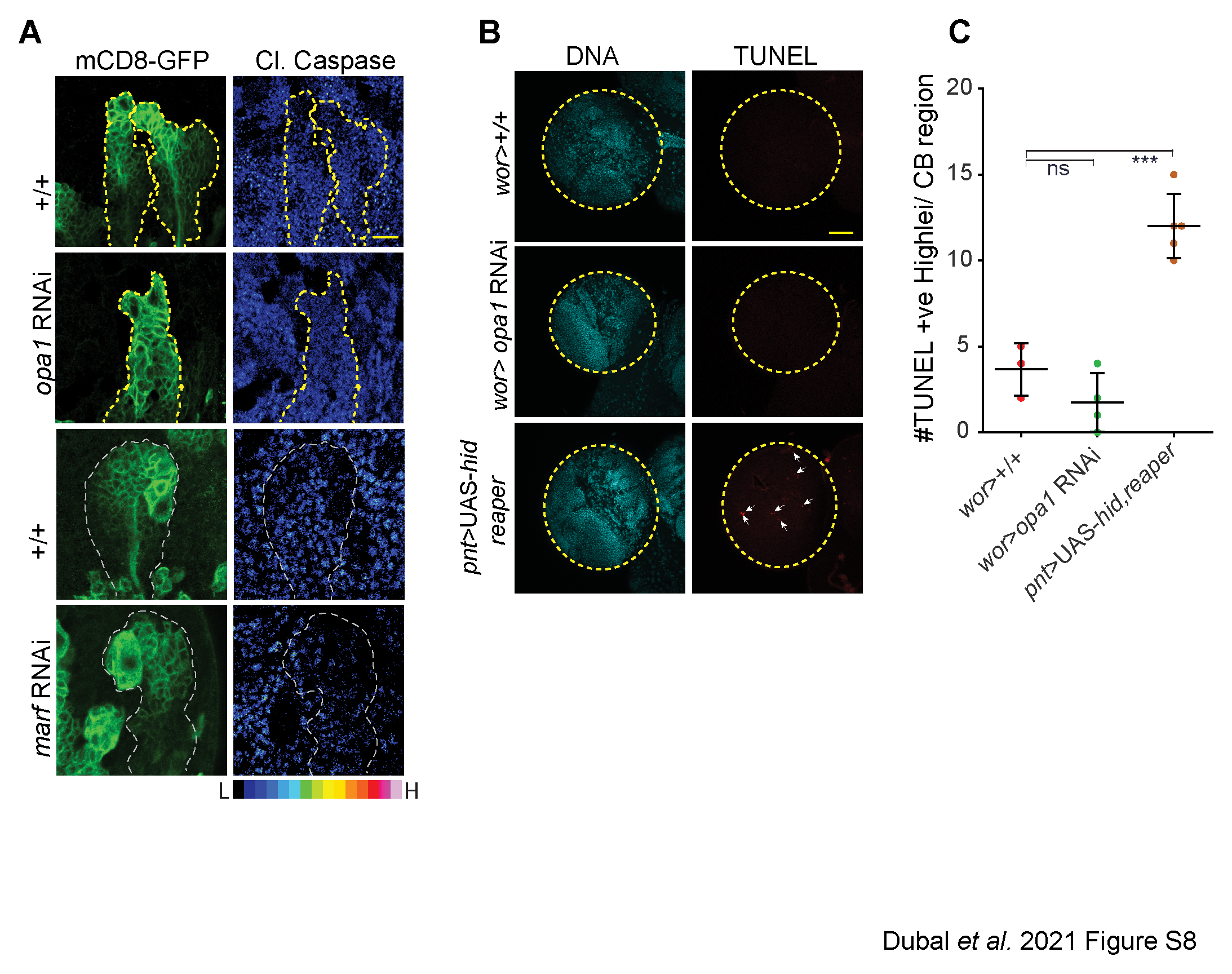

Supplement: S8 Fig — A: Representative images of type II NB lineages (green) stained for cleaved caspase 3 and shown in heatmap. +/+ (for opa1 RNAi, 10 NB lineages, 6 brains), opa1 RNAi (9,6), Control (for marf RNAi, 24 Type II NB lineages, 6 Brains), marf RNAi (30,5). Scale bar- 10μm. B, C: Fluorescence confocal images of brain hemispheres showing no significant change in TUNEL positive nuclei (red, white arrows) (B) in opa1 RNAi expressed in all NBs with wor-Gal4. Scale bar- 50μm. Expression of hid shows a significant increase in TUNEL positive cells when expressed with pnt-Gal4, mCD8-GFP in the type II NB lineage. Quantification of TUNEL positive nuclei (C) in wor-Gal4/+ (3 brains), wor-Gal4 opa1 RNAi (4), pnt-Gal4 UAS-hid reaper (5). Statistical analysis was performed using unpaired t-test. ns- non significant, ***- p<0.0001. (TIF) [file pgen.1010055.s008.tif]

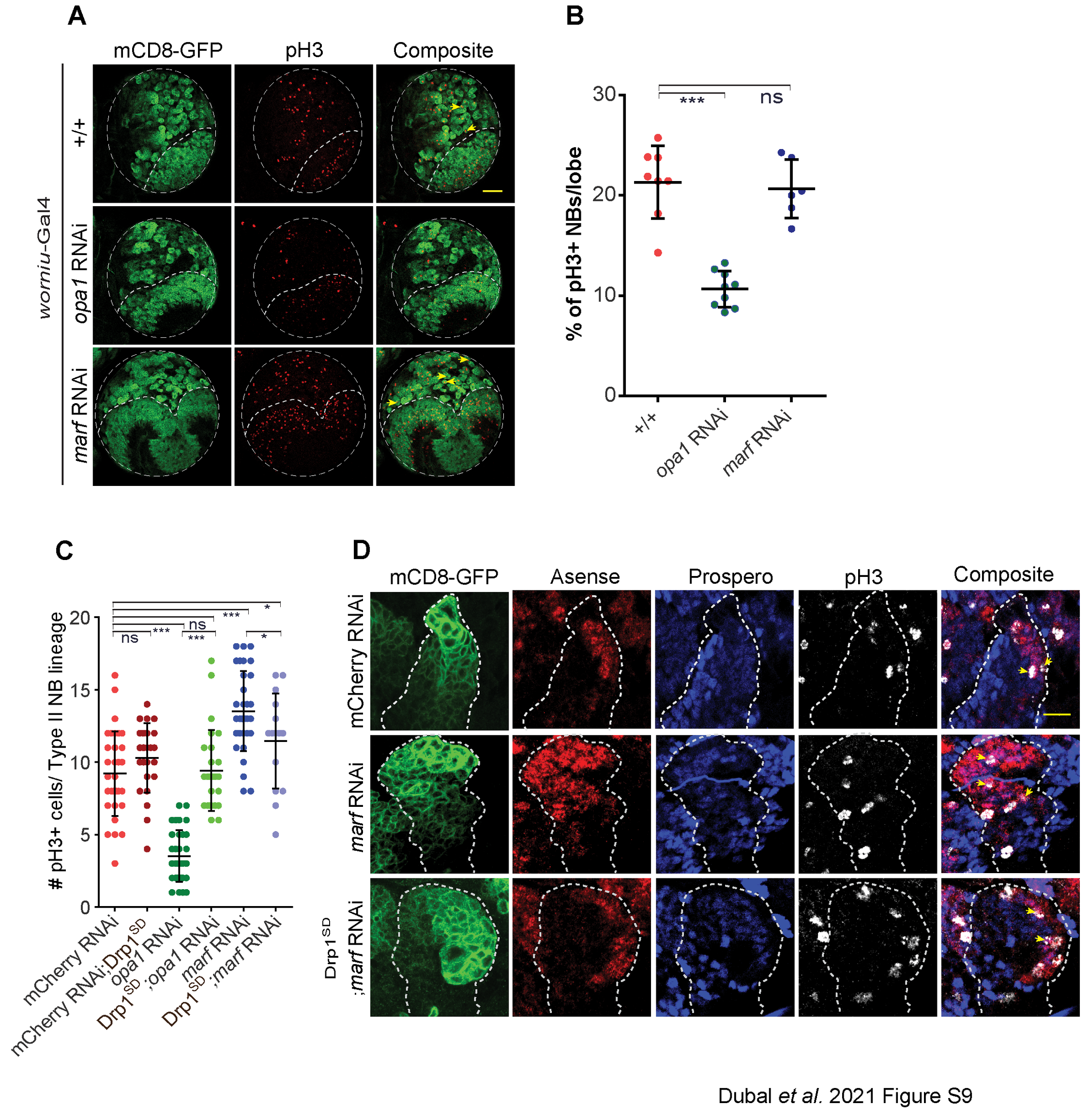

Supplement: S9 Fig — A-B: Representative confocal images of larval brain lobes showing reduction of pH3 positive NBs upon loss of Opa1 (A). Analysis of numbers of pH3 positive NBs (yellow arrows) in larval brain lobe in control (8 brain lobes), opa1 RNAi (9), marf RNAi (6). Scale bar- 50μm. C: Quantification of total number of pH3 positive cells, and percentage of dividing mature INPs in type II NB lineages in mCherry RNAi (33 Type II NB lineages,7 brains), Drp1SD;mCherry RNAi (24,6), opa1 (33,6, Drp1SD;opa1 RNAi (26,8), marf RNAi (33,8), Drp1SD;marf RNAi (15,5). D: Representative images of type II NB lineages labeled with CD8-GFP (green), Pros (blue), Ase (red), pH3 (grey) showing reduced mitotic GMCs (yellow arrows, pH3+light Pros+Ase+) in marf RNAi (in support of graph presented in Fig 5D). Scale bar- 10μm. (TIF) [file pgen.1010055.s009.tif]

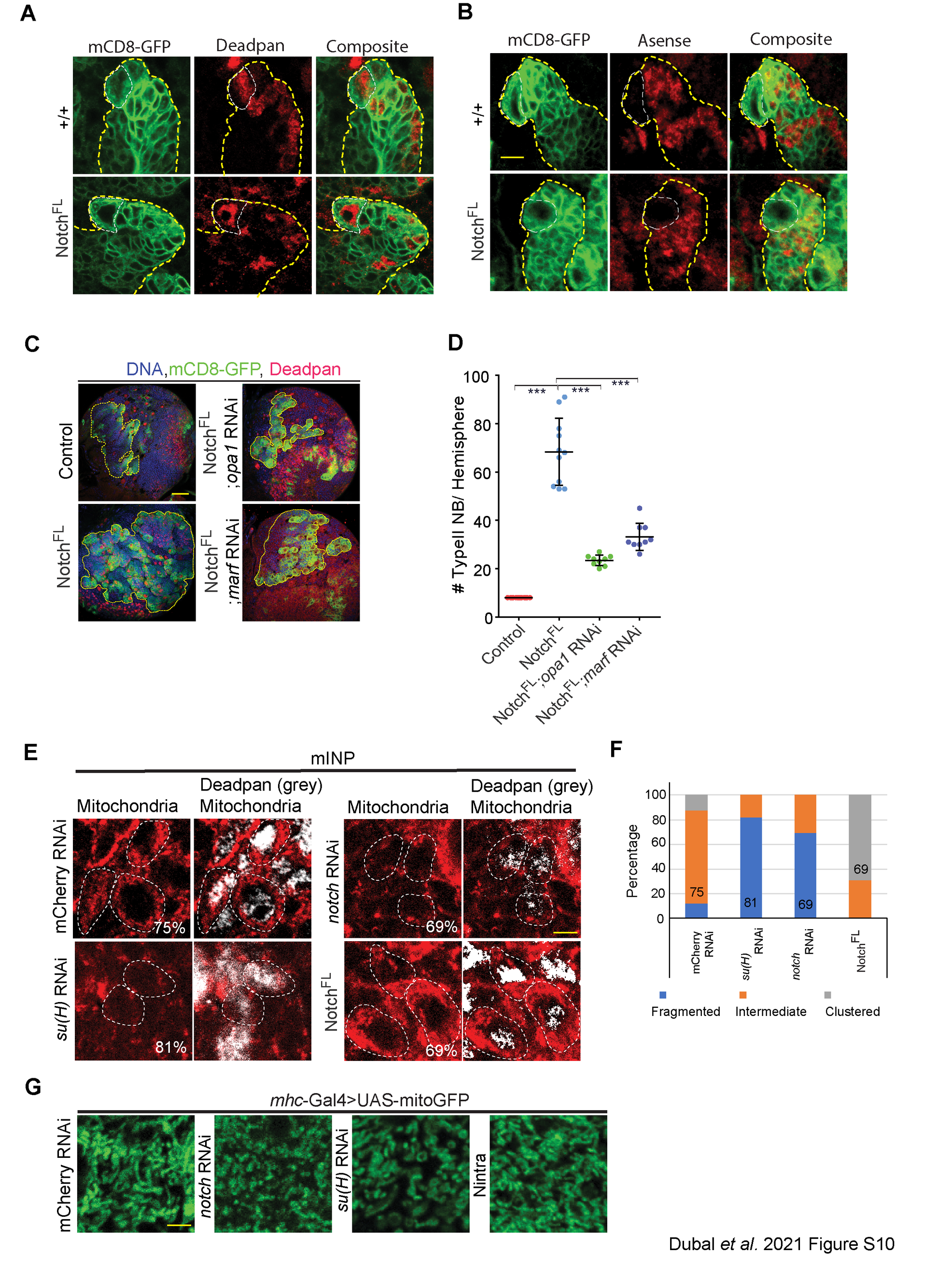

Supplement: S10 Fig — A: Representative confocal images of type II NB lineage (yellow dotted line) showing expression of mCD8-GFP (green) and Dpn (red) in the NBs. Note that Dpn is present in the NB and in the mature INPs in the lineage +/+ (39 Type II NBs, 5 brains), NotchFL (>100, 5). B: Representative confocal images of type II NB lineage (yellow dotted line) showing expression of mCD8-GFP (green) and Ase (red) in the type II NB lineage. Note that Ase is not present in the NB but present in the lineage +/+ (31 Type II NBs, 5 brains), NotchFL (>100, 5). C-D: Larval brain lobes show suppression of Notch mediated NB hyper proliferation on depletion of Opa1 and Marf (C). Quantification of NB number (D) in pnt-Gal4, UAS-mCD8-GFP control (14 lobes), NotchFL (11), NotchFL;opa1 RNAi (9), NotchFL;marf RNAi (9). Scale bar- 50μm. E-F: Confocal images showing mitochondria (red, stained with ATPβ) in mature INPs of type II NBs expressing mCherry RNAi (40 mature INPs, 5 brains, 8 lineages, 75% intermediate), notch RNAi (49 mature INPs, 4 brains, 10 lineages, 69% fragmented), su(H) RNAi (11 mature INPs, 3 brains, 4 lineages, 81% fragmented) and NFL (68 mature INPs, 4 brains, 13 lineages, 69% clustered). The control cohort is the same as that used in Fig 1. Graph shows the distribution of mitochondria into fragmented, intermediate and clustered in the form of a stacked histogram (F). The percentage documented on each bar in the histogram is for the group that is seen at the maximum extent. Scale bar- 2.7μm. G: Representative confocal images of mitochondria labeled with mito-GFP in muscle cells showing smaller mitochondria in notch and su(H) RNAi and larger mitochondria on Nintra overexpression. The image for control mCherry RNAi is repeated from S2 Fig for comparison. Representative images from a minimum of 3 larvae are shown, the phenotype of smaller mitochondria is at 100% in muscle 6 and 7 of segment A2 and A3 in notch and su(H) RNAi and of larger mitochondria in Nintra overexpression. Scale bar-3 [file pgen.1010055.s010.tif]
