# Supplementary material for: Mitochondrial fusion regulates proliferation and differentiation in the type II neuroblast lineage in Drosophila
Source: PLoS Genet. 2022 Feb 14;18(2):e1010055. doi: 10.1371/journal.pgen.1010055 (PMC8880953; doi:10.1371/journal.pgen.1010055)
Supplement: Attachment — Submitted filename: PLOS Genetics Response letter 1.docx [file pgen.1010055.s027.docx]

21^st^ June 2021

**Summary of the changes made in response to the Reviewer comments:**

We thank Reviewer 1, 2 and 3 for careful analysis of the data and writing in our manuscript. We have performed the following revisions in our manuscript in response to the reviewer comments.

1. We have revised the image analysis for mitochondrial morphology from the STED images to obtain area from optical sections which contain discrete mitochondria (Figure 1,3,6, S2).
2. We have obtained the numbers of Dpn, Ase and Pros cells in the type II neuroblast lineage for assessing the change in differentiation (Figure 2,3,7).
3. We have included new controls containing mCherry RNAi (3rd chromosome) or mRFP (2nd chromosome) for comparison with double mutants (Figure 3, S4).
4. We have redone the analysis of pH3 and EdU staining in the type II NB lineage and also added analysis of mitotic mINPs by staining with Dpn and pH3 (Figure 5).
5. We have included new data for the Notch receptor distribution and Notch signaling reporter in the type II neuroblast lineage in different genotypes.
6. We have included Ase staining analysis for the Drp1^SD^; *notch* RNAi combination (Figure 7).
7. We have added Ase staining for NotchFL to assess mINPs (Figure S8).
8. We have focussed our revised manuscript on the role of mitochondrial morphology proteins in neuroblast differentiation rather than the tumor context and discussed this in the response letter.
9. We have revised the flow of the figures to how they appear in text and revised the writing to strictly adhere to the observations in the data.

**The revisions performed to address the comments of each of the reviewers are given below along with a pointwise response to the reviewer comments.**

**Summary of changes made in response to Reviewer 1 comments:**

We thank Reviewer 1 for careful analysis of our data and suggestions of revisions which have helped greatly improve and focus our manuscript to the analysis of differentiation defects in the type II neuroblast lineage on the depletion of mitochondrial morphology proteins. We have made the following changes in the revised manuscript in response to the comments by Reviewer 1.

1. We have revised the image analysis for STED images to assess the mitochondrial area and removed the description of numbers of mitochondria for different genotypes (Figure 1,3,6, S2).
2. We have added new experiments with Ase staining in the type II neuroblast lineage (Figure 2,3,7).
3. We have also added the quantification of immature INPs that do not show the presence of Ase and Pros in the type II neuroblast lineage (Figure 2).
4. We have added the quantification of EdU, pH3 and mitotic mINPs in the proliferation analysis (Figure 5).
5. We have added cleaved caspase staining in the *marf* RNAi (Figure S7).
6. We have revised the description of mitochondrial distribution in type II neuroblasts to be consistent with literature.
7. We now focus our manuscript on the analysis of mitochondrial morphology proteins in type II neuroblast differentiation rather than the tumor context (See response letter Figure 1 for discussion on the tumor neuroblasts).
8. We have added Ase staining in the neuroblast type II lineage expressing NotchFL (Figure S8).

**Reviewer #1:** In this study, Dubal et al. studied potential roles of mitochondria dynamics in normal development of Drosophila type II neuroblast lineages. Specifically, they examined how knocking down mitochondria fusion/fission proteins Opa1, Marf, and Drp1 would affect the morphology of mitochondria in type II neuroblasts, generation of INPs and GMCs. Further, they examined functional relationship between Notch signaling and mitochondria dynamics in type II neuroblast lineage development. Previous studies have shown that oxidative phosphorylation is required for the growth/proliferation of Drosophila tumorigenic neuroblasts, but it seems still a little controversial whether oxidative phosphorylation is required for normal development of type II NB lineages. Therefore, it is interesting to directly examine whether manipulating mitochondrial fission and fusion, which are related with oxidative phosphorylation, would affect normal development of type II NB lineages. However, although this manuscript addresses an interesting question, I have quite a few major concerns that significantly dampen my enthusiasm for this work.

1. A recently published paper (Bonnay F, et al, Cell, 2020, 182: 1490) also examined how knockdown of Marf or Opa1 would affect normal type II neuroblast development and their results showed that knockdown of Marf or Opa1 resulted in a similar or increased number of INPs, which is contradictory to the results described in this manuscript. The authors should address the discrepancy.

**Response:** Bonnay F, et al, Cell, 2020, 182: 1490 have used the RNAi lines for Opa1 and Marf from the VDRC collection, we have not tested these RNAi lines. We have tested Drp1 VDRC lines as compared to Drp1 mutants earlier in the lab and this shows weaker phenotypes as compared to the null allele *drp1*^KG^ or the Drp1^SD^ mutant used here. It is likely that they have weaker phenotypes as compared to the RNAi lines that we have used. Also despite the increase in INPs it is possible that the VDRC lines also show loss of Pros positive neurons and differentiation/proliferation defects in the type II neuroblast lineage, however this remains to be checked in these lines.

1. We have used two different RNAi lines to deplete Opa1 both of which produced similar phenotypes including change in mitochondrial fission, lethality with *worniu-*Gal4 and reduced Dpn, Ase and brightly stained Pros positive cells in the lineage. This observation is also consistent with the reduction in pH3 positive cells and EdU labelled cells in type II NB lineages. Opa1 depletion therefore indeed causes reduction of mature INPs, GMCs and neurons.
2. We have also used two RNAi lines for Marf and they both show no significant difference in Dpn positive mINPs. Ase positive cells increase and brightly stained Pros positive cells at the base of the lineage decrease. There is also an increase in EdU and pH3 positive cells in each lineage on depletion of Marf. There is an increase in mitotic mINPs even though their number does not increase as compared to controls. These data together suggest that GMCs accumulate on Marf depletion and a slowing down of division likely leads to depletion of neurons.
3. It is therefore possible that weaker disruption of Opa1 and Marf causes the phenotype seen in the VDRC lines.

2. Regarding the mitochondria morphology, I am really not sure if anyone could easily see the difference in different groups in Fig 1B, Fig 3A, Fig6C, fig S2A, and FigS5D without looking at the quantification data except that it is clear that expressing Drp1SD leads to aggregation of mitochondria on one side of the NB. They all appear spherical to me. At least I don’t see the clear difference between spherical and elongated/tubular mitochondria structures as shown in some published papers. In fact, mitochondria should mostly appear spherical in normal type I and type II NBs according to published papers rather than tubular as described in this manuscript. The authors may want to confirm their results using different mitochondria markers to verify their findings. Furthermore, the authors should measure the length of the mitochondria for quantifying the mitochondria phenotypes. Also, I am not sure if anyone could count the number of mitochondria accurately with the resolution and quality of the images shown in the manuscript. Personally, I don’t think so and I don’t have confidence in the quantification data of the mitochondrion numbers shown in this manuscript. I don’t think it is extremely important to quantify the number of mitochondria (unless they can truly distinguish individual mitochondria, but that obviously is not the case as they stated that they only quantified the area of “optically resolvable” mitochondria, meaning that at least some mitochondria are not optically resolvable in their images). What matters is the morphology (the area and length) of the mitochondria. If the authors are not totally confident about the quantification data of the mitochondria numbers, these bar graphs should be simply removed.

**Response:** We intended to study mitochondrial morphology using electron microscopy techniques but it has been tough to organise this for sometime now and our institute does not have these facilities on campus. We are extremely interested in addressing mitochondrial morphology with electron microscopy methods in the future. We used STED microscopy as it provides better resolution than confocal microscopy and can be used on neuroblasts which are placed deeper in the brain. We have now carried out the following modifications to the representation of the data from STED images:

1. We have chosen a region in the type II neuroblast which contains discrete distribution of mitochondria and performed a quantification of the area of mitochondrial punctae.
2. As suggested by the reviewer we have removed the quantification of mitochondrial numbers as it indeed does not give us a representation of true mitochondrial numbers in the cell.
3. Based on the new analysis we find that mitochondrial area is decreased in Opa1 and Marf depleted type II neuroblasts and increased in Drp1^SD^ mutants. In addition, Drp1^SD^ expressing neuroblasts show a cluster of mitochondria on one side of the neuroblast.

3. The authors counted wrong cells for quantifying the number of GMCS for Fig 2E, Fig 3G, Fig 7E, Fig S2G, Fig S3F. The authors used Pros staining as a single marker for identifying GMCs. However, Pros is not only expressed in GMCs but also neurons (particularly young neurons). The expression of nuclear Pros in GMCs is usually weaker than that in neurons. The authors counted mostly the cells with strong Pros staining as GMCs, but actually these cells mostly are neurons rather than GMCs. To quantify GMCs, they need to use additional makers such Elav or Ase. GMCs express nuclear Pros and Ase but not Elav or Dpn. In addition, I don’t think their quantification of the number of immature INPs in fig 2C is totally accurate. The number of immature INPs never exceed 10 per lineage in normal brains, but they have several samples that have more than 10 imINPs per lineages. The authors have to make sure not to include cells with weak nuclear Pros staining as imINPs or they could other markers such PntP1 staining to label imINPs. Further, even if the authors use the Pros staining for quantifying the number of neurons, the quantification results may not be reliable either because mCD8-GFP driven by pntP1-GAL4 can not label all cells in type II NB lineages.

**Response:** We thank the reviewer for this discussion. We have redone the entire quantification after obtaining the rat-Ase antibody from the Knoblich lab for this revision. We did obtain other antibodies from the Jan lab as well to perform a double staining for Ase and Dpn, however they did not survive the shipment, hence we were not able to perform double immunostainings for Dpn and Ase simultaneously.

We summarise the identity of the cells quantified in Figure 1A based on literature, cells negative for Ase and Pros adjacent to the type II NB are immature INPs, Dpn positive cells are mINPs, Ase positive cells are immature INPs, mINPs and GMCs and brightly stained Pros positive cells at the base of the lineage are neurons.

We are staining the *pnt*P1-Gal4, UAS-mCD8-GFP brains with chicken anti-GFP to be able to discern the anti-GFP signal throughout the lineage, especially at the base. The *pnt*P1-Gal4, UAS-mCD8-GFP highlights cells at the base of the lineage, these are lighter in GFP intensity than the mINPs and GMCs, which are spatially closer to the neuroblast. We have counted all the Pros positive neurons that are the base of the lineage, and are positive for the mCD8-GFP signal and brightly stained for Pros. We are able to get a consistent reproducible number replicates between the various different controls used in the manuscript.

Based on the analysis of our new staining we find the following:

1. Opa1 depletion leads to a decrease in Dpn, Ase and brightly stained Pros positive cells at the base of the lineage. This together shows that there is a depletion of mINPs, GMCs and neurons in Opa1 depleted type II neuroblast lineages.
2. Marf depletion leads to a decrease in brightly stained Pros positive cells at the base of the lineage. The Dpn positive cells are almost similar in numbers to controls and the Ase positive cells are increased. This together shows that there is a loss of differentiation from the GMC to neurons in the type II neuroblast lineage on depletion of Marf.
3. Drp1^SD^ expressing type II neuroblast lineages do not show a defect in the numbers of cells in the lineage.

4. The authors showed that Marf RNAi knockdown led to a reduction in the number of Pros+ cells in type II NB lineages but did not affect the number of imINPs, mINPs, or pH3+ cells in the lineages. Logically it does not make sense unless that there is an increase in apoptotic cell death of postmitotic neurons, which seems not likely based on what they found with Opa1 knockdown. The authors may want to take a look at apoptosis after knocking down Marf and discuss why there is a reduction in Pros+ cells after knocking down Marf.

**Response:** We performed the following revisions to address these comments:

1. We re-quantified Dpn+ and pH3+ data in *marf* RNAi along with other genotypes. We find that the number of pH3+ cells increased while Dpn+ cells remained constant.
2. We performed co-staining of Dpn and pH3 to identify cell types that are undergoing division. We found an increased number of cells that are positive for both Dpn and pH3 in type II neuroblast lineages depleted of Marf.
3. We immunostained brains with Asense which marks both mature INPs and GMCs. Ase+ cells are also increased in Marf depletion. Together this data indicates that Marf depletion causes lowered proliferation of GMC population which further results in reduction of post-mitotic neurons (brightly Pros^nucl^ stained cells). Reduced proliferation may be the reason for accumulation of GMCs in the lineage.
4. Further to confirm depletion of Pros positive cells in *marf* RNAi is not due to the apoptosis in the lineage, we performed cleaved caspase-3 staining in control and *marf* depleted type II lineages. Cleaved caspase-3 levels in the lineage were similar to controls in *marf* RNAi suggesting that the loss of cells was not a result of increased apoptosis.

5. In Fig 6, the data show that knockdown of Opa1 leads to a reduction in the number of pH3+ cells in type II NB lineages but expressing Drp1SD does not, however, when Opa1 RNAi and Drp1SD are expressed simultaneously, there is an increase in the number of pH3+ cells. Logically it does not make sense. Same concern for the increased number of pH3+ cells resulting from the simultaneous expression of Marf RNAi and Drp1SD. The authors may want to double check their quantification data to make sure they are accurate.

**Response:** As suggested by the reviewer, we repeated our pH3 quantification. We also add EdU staining for all the genotypes. In addition to this, we performed co-staining of pH3 and Dpn to mark dividing mature INPs in the lineage. We found that dividing mINPs (Dpn+ pH3+) are reduced in Opa1 RNAi while they are unchanged in Drp1^SD^. But co-expression of Opa1 RNAi and Drp1^SD^ rescued mINP number. The proportion of cells which are dividing in Opa1 RNAi expressing type II neuroblast lineages also decreased. This in addition to the decrease in EdU positive cells shows that there is a decrease in proliferation of the mINPs and possibly also GMCs on Opa1 depletion. This phenotype of decrease in proliferation was suppressed when Opa1 and Drp1 were depleted together. The mitochondrial defect of increased fragmentation is suppressed in Drp1 and the differentiation defect of loss of mINPs is reversed. This shows that the defect in Opa1 depletion caused by mitochondrial fragmentation was suppressed when mitochondria were fused in the Drp1^SD^;*opa1* RNAi combination.

Interestingly Marf depletion showed an increased number of mitotic mINPs which were rescued in Drp1^SD^; *marf* RNAi combination. Marf depletion showed increased EdU labeled cells which are rescued in Drp1^SD^; *marf* RNAi combination. The brightly stained Pros positive neurons were however decreased in *marf* RNAi expressing neuroblasts. Since mINPs did not increase, GMCs increased and Pros positive neurons decreased on Marf depletion, this suggests a delay in exit from mitosis in mINPs.

6. I do not find data shown in Fig 6 are particularly informative. The purpose of this figure is to investigate why knocking down of Opa1 or Marf leads to reduction in the number of mINPs and/or Pros+ cells. They need to look at the mitotic rate of individual cells types including type II NBs, mINPs, and GMCs separately rather than just count the total number of pH3+ cells in the lineages, which does not tell whether there is any defect in proliferation of type II NBs, mINPs or GMCs. With the data they have right now, they cannot draw the conclusion that “opa1 depletion decreased the rate of mINP proliferation” (at the end of 1st paragraph on page 11).

**Response:** As suggested by the reviewer we have now added data addressing the mitotic rate of the mINPs in different genetic combinations. We find that depletion of Opa1 causes decrease in the numbers of mitotic mINPs while Marf depletion leads to an increase in these numbers. This data has been added to revised Figure 5.

7. There is inconsistency between what the authors described and the literature they cited. For example, at the beginning of the second paragraph on page 5, they described that mitochondria are tubular and cited the reference #31. However, in that reference, it is clearly stated that mitochondria in NBs appear spherical, which is also confirmed in other publications such as the reference #24 they cited.

**Response:** We thank the reviewer for discussing the description of mitochondrial morphology in order to make it consistent with literature. Mitochondria appear bead-like spheres in controls and are present as discrete spheres in mutants. The morphology of mitochondria in type II neuroblasts appears similar to the previously described morphology in type I neuroblasts.

8. In Fig S3 (the 3rd paragraph on page 6), the authors used woriu-GAL4 to knockdown Opa1, Marf, or Drp1 and labeled the brains with Miranda staining for quantifying the number of type I and type II NBs. Miranda is expressed in both type I and type II NBs. Without any additional markers, how could the authors distinguish type I vs type II NBs?

**Response:** We do not address differences between type I and type II neuroblasts by this experiment. We have performed the experiment with *worniu* Gal4 to assess if both type I and type II neuroblast lineages show defects in mitochondrial morphology mutants. We find that there is no change in numbers of neuroblasts labelled with Miranda when depleted of mitochondrial morphology proteins.

9. For Fig S4A-B, I am not convinced that there is any positive pAMPK staining signal in the NBs. They all appear similar to the background.

**Response:** pAMPK levels elevate in ATP deprived cells or in mitotic cells [[1]](https://paperpile.com/c/tIJBWg/gyfT). We have observed an increase in pAMPK in the cytoplasm in electron transport chain mutants but not mitochondrial morphology mutants in our previous studies in embryogenesis [[2,3]](https://paperpile.com/c/tIJBWg/ydj4+62A1). In the images we can see mitotic cells strongly labelled with pAMPK. However we did not observe a change in pAMPK signal in control and Opa1 RNAi which indicates that there is no ATP stress in Opa1 depleted NBs. For a positive control we treated brains with glycolysis inhibitor, 2-DG, and since treatment is general and not specific to particular cells, we could observe a tissue wide increase in the pAMPK signal which is reflected by increased gray levels on imaging DMSO and 2-DG treated samples at the time under the same laser settings. For better representation we have changed pAMPK images to gray scale and updated them in the revised manuscript.

10. In Fig 6A-B, the authors quantified the number of mINPs by counting the number of Dpn+ cells. However, expressing NotchFL would lead to de-differentiation of imINPs and generation of extra type II NBs. They need to include additional Ase staining to ensure they are not counting the dedifferentiated type II NBs after expressing NotchFL.

**Response:** We have now focussed our manuscript on the analysis of the role of mitochondrial fusion in type II neuroblast differentiation rather than the tumor context. Since we are not able to address the role of mitochondrial morphology on neuroblast dedifferentiation, we have only included this data for discussion in the response letter.

As suggested by the reviewer, we show that the NotchFL brains at the third instar larval stage contain type II neuroblasts positive for Dpn and negative for Ase (Figure S8). These are larger cells at the head of the lineage. We stain these brains with ATPb to observe mitochondrial morphology in them and find that mitochondria appear clustered like the *drp1* mutants. This result is also consistent with the observations of fused mitochondria in Brat and numb RNAi in recent reports [[4]](https://paperpile.com/c/tIJBWg/0JZM).

11. In Fig 6C-E, the authors found that expressing NotchFL led to an increase in the mitochondria area and a reduction in the number of mitochondria. However, when Nintra was express, they found that the area of mitochondria was not affected but the number was reduced. Nintra is supposed to have stronger effect than NotchFL. It has been shown in the reference #24 that knocking down Numb, which will lead to over-activation of Notch signaling like the expression of Nintra, led to fusion of mitochondria and that this phenotype is a common feature of tumorigenic NBs as knockdown of Brat also led to a similar fusion of mitochondria. Therefore, the results are contradictory to published data. It seems unlikely that expressing Nintra would not lead to tubular mitochondrial morphology and an increase in the area of mitochondria.

**Response:** We have revised the mitochondrial area quantification in the entire manuscript by using a region in the central optical plane of the neuroblast containing discrete mitochondria. The repeated quantification shows that mitochondrial area is in fact significantly increased in Nintra expressing NBs to that of control. This result is also consistent with elongated mitochondria seen in Brat RNAi in recent reports [[4]](https://paperpile.com/c/tIJBWg/0JZM).

12. The loss of mINPs in Notch RNAi type II NB lineages is at least in part to due to ectopic Ase expression in type II NBs and subsequent transformation of type II NBs into type I NB. If Drp1SD expression partially rescued the number of mINPs and Pros+ cells in Notch RNAi type II NB lineages as shown in Fig 7, does the Drp1SD expression also suppress the ectopic Ase expression in Notch RNAi type II NBs? If mitochondrial fusion induced by the expression of Drp1SD could not rescue the loss of type II NBs resulting from Notch RNAi, why could it alleviate notch signaling defects in mINPs and rescue the loss of mINPs? Could the author provide any reasonable explanations?

Also, in Fig 7D, it seems that Pros staining did not work well for Drp1SD Notch RNAi group. The staining pattern is very different from those in other panels. Dpn staining for NotchFL; *marf* RNAi in Fig7F did not go well either in Fig7F. It looks like just background. The author should redo the staining of Pros and Dpn for these two groups.

Further, co-staining of Ase should be done for Fig7F to unambiguously distinguish type II NBs from mINPs because the expression of NotchFL causes dramatic increase in the number of type II NBs due to dedifferentiation of imINPs and some dedifferentiated NB may still remain small and are difficulty to be distinguished from mINPs based on the cell size.

**Response:** We thank the reviewer for this discussion. To address these comments we have performed new experiments and included the following data:

1. We performed a staining of Dpn, Ase and Pros in Drp1^SD^; *notch* RNAi brains and compared them to *notch* RNAi alone. We show that in Drp1^SD^; *notch* RNAi there is a suppression of loss of Dpn positive cells in each lineage, partial suppression of the loss of Ase positive cells and no change in the loss of Pros positive neurons (Figure 7).
2. Ase is not present in the type II neuroblast lineages at the third instar larval stage in Drp1^SD^; *notch* RNAi (Figure S8). Ase has been found in Notch depleted neuroblasts at earlier stages which convert to type I according to literature [[5]](https://paperpile.com/c/tIJBWg/YHkN).
3. We have Dpn and Ase antibodies in the same animal and hence we could not check for loss of conversion to type I by dissecting brains at earlier time points. We have analysed lineages at the third instar larval stage and we see that Dpn positive cell numbers revert back to control numbers.
4. Mitochondrial morphology does not affect the numbers of type II neuroblasts in controls and in Drp1^SD^; *notch* RNAi combinations. Drp1^SD^ expression did not suppress the defect in proliferation of the type II neuroblasts depleted of *notch* RNAi per se but only the mINPs in the lineage. Loss of Notch signaling converts type II neuroblasts to type I neuroblasts and it is likely that inhibition of Drp1 function is not strong enough to suppress this conversion. Loss of fusion by Opa1 depletion affects Notch in the neuroblast and in the lineage but it appears that the extent of depletion is not sufficient for conversion to type I neuroblasts. The type II lineages that form in *notch* RNAi show a decrease of Dpn, Ase and Pros positive cells, this phenotype is partially suppressed by Drp1 mutants.
5. We have now removed the analysis of tumor neuroblasts from the manuscript. We retain the preliminary data obtained on this is in the response letter (Response letter Figure 1, page 18). As suggested by the reviewer, we have performed immunostaining of Ase in NotchFL and NotchFL; *marf* RNAi/*opa1* RNAi. We find that the larger cells which are type II neuroblasts are negative for Ase. These cells are decreased on depletion of Marf and Opa. We have not been able to perform a Dpn and Ase costaining as they were raised in the same animal.

13. The authors need to describe clearly what they mean by “differentiation of type II NB lineages” when they use this term to describe the phenotypes. This is a very vague term and could mean a lot of things, such as differentiation of NBs, differentiation of INPs, neuronal identity specification, etc. They need to be more specific when describing the phenotypes. They need to make it clear the phenotypes are defects in type II NB proliferation/self-renewal, INP maturation/proliferation/self-renewal, GMC production/division, or neuronal production/fate specification. The authors also should not use the term “mutant” for RNAi knockdown. They are different.

**Response:** We thank the reviewer for this discussion, we have now concluded that mitochondrial fusion is needed for differentiation in the type II neuroblast lineage based on the following data.

1. There is no change in the numbers of type II neuroblasts on depletion of Opa1 and Marf
2. The numbers of immature INPs negative for Ase and Pros do not change in mitochondrial morphology mutants.
3. mINPs, GMCs and neurons decrease on depletion of Opa1.
4. GMCs increase and neurons decrease on depletion of Marf.
5. Drp1 mutants do not show a defect in differentiation in cells in the type II neuroblast lineage.

14. The subtitle on page 12 “Mitochondrial fusion induces differentiation on Notch depletion in the type II NB lineage” is very confusing. Did the author mean “Mitochondrial fusion induces differentiation of Notch-depleted type II NB lineages”?

**Response:** As suggested the subtitle is changed to “Mitochondrial fusion induces differentiation of Notch-depleted type II NB lineages” in the revised manuscript.

**Summary of changes made in response to Reviewer 2 comments:**

We thank the reviewer 2 for a thorough reading and comments on our manuscript. We summarise here the changes made specifically in response to comments by reviewer 2.

1. We have revised the method of analysis of mitochondrial area from STED microscopy and added this description to the methods section (Figure 1,3,6, S2).
2. We have focussed the manuscript on the role of mitochondrial morphology proteins in type II neuroblast differentiation rather than the tumor context (See response letter figure 1 for discussion on this).
3. We have added literature on the use of the RNAi and mutants in previous studies.
4. We have added new data on analysis of cells in the type II neuroblast lineage with labelling of Dpn, Ase and Pros (Figure 2,3,7).
5. We have changed the organisation of the figures to match the writing and revised the language of the figures to stick to interpretations from the data.

**Pointwise Response to comments by Reviewer 2**

**Reviewer #2:** Dubal et al., shows in this ms that mitochondria architecture is important for differentiation of type II lineages. They show that Drp1 (fission protein) has a different phenotype to the of Opa1 and Marf (inner membrane fusion protein and out membrane protein) respectively. The latter two shows loss of mitochondria activity and failure for type II NBs to differentiate (just like notch mutant). OPa1 is also required for MMP and caused increased cytochrome c in type II NBs when inhibited. Opa1 depletion decreased the rate of mINP proliferation in the type II NB via decreasing its rate of cell cycle progression. Finally, the authors show that inhibiting Notch RNAi causes failure to differentiate, and this can be rescued by Drp1 dominant negative. The work is of general interests to the field of developmental neurobiology, as it shows that fission and fusion of the mitochondria is important for NB differentiation.

Major points:

I see two main issues with this ms. First is the quantification of mitochondria fusion/fission phenotype, the images are not very obvious to interpret, and how quantification can reflect these phenotypes is not so clear. Either representative 3D reconstruction should be provided, or clearer explanation of how quantification is done should be provided.

**Response:** We have wanted to do electron microscopy analysis for assessing the mitochondrial morphology in controls and mitochondrial morphology mutants. We do not have electron microscopy facilities on our campus and it has been impossible to travel for this for a while now.

As suggested by the reviewer 1, we have now improved our quantification using STED microscopy. STED gives better resolution as compared to confocal microscopy and can be performed on cells deeper in the brain. We have now redone the quantification on central sections and in regions which contain discrete mitochondria. We have computed the area of the mitochondria in controls and mutants. We have added the method for this in the methods section. As pointed out by reviewer 1 we have removed the quantification of mitochondrial numbers since it is impossible to resolve all the mitochondrial structures in this analysis and find out the actual total numbers of mitochondria in the cell. We find that the mitochondrial area is reduced on Opa1 and Marf depletion as compared to controls and increased in Drp1 mutants. In addition Drp1 mutants show mitochondria organised as a cluster on one side in the type II neuroblast. Mitochondrial area is also increased in NotchFL and NICD overexpression and reduced in *notch* RNAi and Su(H) RNAi in the type II NBs.

The second issue is regarding the discrepancy of Notch activated vs Notch RNAi phenotype, and why Notch intra/Full length is rescued by Opa1 and Marf in terms of NB number, and conversely why Drp1 DN rescues the differentiation phenotype (which makes a lot more sense than the former).

**Response:** The depletion of mitochondrial fusion proteins does not have an effect on neuroblast numbers. There is a loss of differentiation in the type II neuroblast lineage and Pros positive neurons are decreased in each lineage. There is a decrease in mitotic mINPs and loss of Notch signaling in the type II neuroblast lineage on Opa1 depletion. Combining the Drp1 mutant with *notch* RNAi leads to suppression of loss of Dpn and Ase cells in the Opa1 depleted type II lineage. It does not lead to suppression in the loss of numbers in the type II neuroblasts.

We have now removed the analysis of Opa1 and Marf depletion in the context of tumor NBs to focus our study on differentiation. These data are a part of Response letter Figure 1 for discussion. The proliferation of tumor neuroblasts formed due to increase in Notch signalling by overexpressing NICD or NotchFL is dependent upon mitochondrial fusion proteins Opa1 and Marf. Recent studies show that mitochondrial fusion is required for proliferation of brat and numb RNAi tumor neuroblasts also [[4]](https://paperpile.com/c/tIJBWg/0JZM). We reason that this is likely because loss of Opa1 and Marf leads to a defect in conversion of mINPs to type II neuroblasts. We are pursuing this question in a future study.

Our analysis shows that loss of Opa1 and Marf along with NotchFL leads to decrease in Pros positive cells in the lineage. The neuroblasts at the third instar larval stage do not show the presence of Ase (revised Figure S8). This loss of proliferation is also seen along with NICD. Since this result of loss of Pros positive cells on depletion of Opa1 and Marf is similar to what we have seen in the condition of Opa1 and Marf depletion in controls and the analysis of dedifferentiation requires in depth investigation, we are removing this data from the manuscript in the revised version.

1. “Opa1, Marf and Drp1 depletion by multiple RNAi lines and mutants showed survival of animals until the pupal stage with inscuteableGal4 and worniu-Gal4 and were lethal or showed behavioral defects as adults.”MarfRNAi seems to be published else where, is Opa1 and Drp1 RNAis validated? Should be done with qPCR.

**Response:**

As noted by the reviewer, these mutants have been used in previous studies by analysis of mitochondria using fluorescence studies and with electron microscopy.

The *opa1* RNAi and *marf* RNAi have been used earlier and loss of mitochondrial fusion has been shown by electron microscopy analysis. *opa1* RNAi (BL32358): [[6]](https://paperpile.com/c/tIJBWg/XX11)[[7]](https://paperpile.com/c/tIJBWg/7J2z)*, marf* RNAi: [[8]](https://paperpile.com/c/tIJBWg/4TrL) [[9]](https://paperpile.com/c/tIJBWg/lOXD)

Drp1 mutants have been used extensively earlier. *drp1*^KG^, a null mutant of Drp1, has been used earlier in neurons, spermatocytes and follicle cells in previous studies [[10–13]](https://paperpile.com/c/tIJBWg/PYmj+QWrY+XZAl+Xwe3). Drp1S193 mutant used in this study has been used earlier in embryos to show mitochondrial fusion and defects in embryogenesis [[2]](https://paperpile.com/c/tIJBWg/ydj4).

Mitochondrial clustering on Drp1 depletion has been seen earlier in mammalian cells [[14,15]](https://paperpile.com/c/tIJBWg/m1Yp+m3v8) and also in *Drosophila* spermatocytes, neurons, follicle cells and embryos depleted with Drp1 [[2,11–13,16]](https://paperpile.com/c/tIJBWg/XZAl+QWrY+Xwe3+hliT+ydj4). Mitochondrial clusters have been observed to have inter connected mitochondrial tubes in electron microscopy studies in mammalian cells [[15]](https://paperpile.com/c/tIJBWg/m3v8).

We are also generating HA tagged lines for Drp1, Marf and Opa1 with endogenous promoters in combination with the mutants to test the levels of these proteins in a lineage specific manner in controls, RNAi knockdown and Notch mutants and overexpression. We will make this analysis a part of a future study.

2. It is not clear to me how mitochondria number is estimated in Fig 1B and S2, as the mitochondria punctas are not well separated. In the methods, it is stated that watershed tool is used, however, does this work, if all objects are touching? Additional more convincing images are needed to support this data.

**Response:** We have repeated the mitochondrial area quantification and removed the use of the watershed tool. In the new analysis, we have performed quantification of mitochondria in central optical planes of the type II NB and in areas which contain discrete mitochondria. We selected a fixed region of interest where mitochondrial punctae are resolved followed by intensity threshold based selection of mitochondrial punctae. Then we quantified the area of each puncta by keeping a lower size cut off of 0.12 um^2^. Next we calculated the average area of all punctae present in the given ROI. For this analysis we have kept ROI sizes the same for all the genotypes and did not apply the water shedding tool. We have added the updated graphs and analysis description in the revised manuscript. It is true that some mitochondria will touch each other and will not be resolved but in general this methods can be used effectively and consistently to show the difference between controls and Opa1/Marf depletion.

3. What are the weakly positive pros cells in Figure 2 S2 and Figure 3? How do you make conclusions that it is GMCs and not INPs, if Dpn is not used in conjunction in the same sample?

**Response:** The lighter Pros positive cells are mINPs and GMCs. To avoid the confusion between mature INPs and GMCs, we have separately performed Asense (Ase) staining which marks both the cell types. Since Ase is present in a few immature INPs, mature INPs and GMCs while Dpn marks only mature INPs, the numbers of Ase+ cells are greater than Dpn+ cells.

We could not perform co-staining of Ase and Dpn together because both the antibodies were raised in the same animal (rat).

We have added this data in the revised manuscript.

4. Notch RNAi causes mitochondria fragmentation and by suppressing Drp1, then differentiation (not nb number) is rescued significantly. To better quantify the differentiation phenotype, overall clone volume of type II lineages under the genetic manipulations should be estimated.

**Response:**

We have added a quantification for *notch* RNAi with Drp1^SD^ for Dpn, Ase and Pros positive cells in the type II neuroblast lineage. We attempted the clone volume analysis but were not able to add this since the lineages merge at the bottom and also become lighter. The Pros positive cells are at the base of the lineage and we have quantified these for the mitochondrial morphology depletions and the suppressions.

5. TMRM intensities were computed in the entire type II NB in control, opa1 RNAi and marf RNAi expressing brains and in the clustered mitochondria in Drp1SD, Drp1SD;opa1 RNAi and Drp1SD;marf RNAi expressing brains using ImageJ. Why different parameters?

**Response:** As suggested by the reviewer, we have repeated the quantification by selecting only TMRM intensities using the threshold tool and re-plotted the graph and updated it in the revised manuscript. The intensities were always computed as an average per pixel.

6. Notch intra full length gives ectopic NBs, and Opa1 and Marf1 RNAi surpresses the fusion, and somehow affects NB number. How this happens is not clear to me. The authors suggests it is via affecting NB proliferation, but it could also be due to affecting the rate of INP to NB dedifferentiation, the authors should show which is the case.

**Response:** We agree with the reviewer that this could be occurring due to an effect on INP dedifferentiation. We find that Ase is not expressed in the neuroblast in the NotchFL; *opa1*/*marf* RNAi combination (Response letter figure 1). However we do not have a Dpn and Ase antibody raised in different animals and hence were not able to execute a clear experiment to distinguish between the possibilities of lack of dedifferentiation or an impact on proliferation. Mitochondrial fusion has been recently found to be important for tumor neuroblasts formed *brat* RNAi [[4]](https://paperpile.com/c/tIJBWg/0JZM).

In this manuscript we focus on understanding the role of mitochondrial morphology proteins in neuroblast different rather than the tumor context. In our analysis depletion of mitochondrial fusion proteins does not change neuroblast numbers. We therefore have moved our preliminary data of loss of tumor proliferation in neuroblasts on depletion of mitochondrial fusion proteins out of the current version of the manuscript. This is a part of the Response letter figure 2.

Response letter Figure 1: A-B: Larval brain lobes show suppression of Notch mediated NB hyper proliferation on depletion of *opa1* and *marf* (A). Quantification of NB number (B) in *pnt*-Gal4, UAS-mCD8-GFP control (14 lobes), Notch^FL^ (11), Notch^FL^;*opa1* RNAi (9), Notch^FL^;*marf* RNAi (9). Scale bar- 50μm

C-D: Larval brain lobes (mCD8-GFP, green) increase in size on Nintra overexpression which is rescued by *opa1* RNAi and *marf* RNAi expression. Representative images of larval brain hemisphere (B, Top, scale bar- 50μm) containing mCD8-GFP (green) DNA (blue) and Dpn (red) showing partial rescue of NB hyperproliferation in Nintra expression by *opa1* RNAi and *marf* RNAi (B). Increased Brain size in Nintra is rescued by *opa1* RNAi and *marf* RNAi expression (B, Bottom, scale bar- 200μm). Nintra (8 lobes, 8 brains), Nintra;*opa1* RNAi (10,10), Nintra; *marf* RNAi (10,10). Scale bar- 50μm. Quantification of lobe size (D) in control (8 brain lobes), Nintra (12), Nintra;*opa1* RNAi (10), Nintra; *marf* RNAi (12). Graph shows mean ± sd. Statistical analysis was performed using unpaired t-test. *-p<0.05, ***- p<0.001.

E: Representative confocal images of type II NB lineages showing expression of mCD8-GFP (green), Ase (red) and Pros (grey). Notch^FL^ (>100 Type II NB lineages, 5 Brains), Notch^FL^;*opa1* RNAi (>100, 5), Notch^FL^;*marf* RNAi (>100, 5). Scale bar- 10μm

F: Representative superresolution STED images of mitochondria showing clustered morphology in Nintra mutant expressed with *pnt*-Gal4, UAS-mCD8-GFP and punctate appearance in *opa1* and *marf* RNAi expressing type II NBs in the Nintra background. Nintra (76% tubular, 58 Neuroblasts, 6 Brains), Nintra;*opa1* RNAi (100%, 95, 20), Nintra*;marf* RNAi (100%, 98, 14). Scale bar- 10μm

It is possible that Notch driven dedifferentiation of mINPs to neuroblasts is inhibited by depletion of mitochondria fusion proteins in the tumor context.

Minor points:

S3 was cited before S2 D,E, citing of S figures are all over the place.

**Response:** As suggested by the reviewer we have changed the organisation of all the figures to match the flow of the writing.

“Our data together shows that mitochondrial morphology is tubular in type II NBs and fusion of mitochondria alone did not give a differentiation defect but was able to suppress the differentiation defect in opa1 mutant NBs”. This is a long sentence, perhaps needs rewriting.

**Response:** We have re-written the statement as follows: Our data shows that mitochondrial fusion on Drp1^SD^ overexpression did not have any differentiation defects and it also suppressed the differentiation defect in *opa1* depleted type II NB lineages.

**Summary of changes made in response to Reviewer 3 comments:**

We thank the reviewer 3 for thorough analysis of our data and the writing in the manuscript. We have made the following changes in response to the reviewer 3 comments:

1. We have added more controls such as mCherry RNAi (3rd chromosome) along with Drp1^SD^ and mRFP (second chromosome) along with *opa1* RNAi and *marf* RNAi in the manuscript for assessing the dilution of the pointed-Gal4 in suppression experiments (Figure 3, S4).
2. We have made changes to the analysis from STED images for mitochondrial morphology (Figure 1,3,6, S2).
3. We have compared mitochondrial distribution in Drp1^SD^ mutants to the previously characterised null alleles of *drp1*^KG^ and added literature that notes mitochondrial clustering on depletion of mitochondrial fission protein Drp1 (FIgure S2).
4. We have added the data on Notch receptor trafficking and Espl-GFP levels for assessing Notch signaling levels in mitochondrial mutant type II neuroblasts (Figure 6).
5. We have changed the language and flow of the figures as recommended by the reviewer.
6. We have revised the data in this manuscript to focus on the function of mitochondrial morphology proteins in differentiation in the type II neuroblast lineage rather than the tumor context (see response letter figure 1 for discussion and data on the tumor context).

**Pointwise response to Reviewer 3 comments**

**Reviewer #3:** In this manuscript, Dubal et al investigated the role of mitochondrial morphology during differentiation of type II neuroblasts in Drosophila. The authors performed genetic and imaging experiments to assess the effect of blocking mitochondrial fusion or fission on neuroblast proliferation, differentiation and viability. They found that depletion of Opa1 results in changes in mitochondrial network and reduced neuroblast differentiation. The authors further showed the differentiation defect induced by knocking down Opa1 is not due to cell death, but more likely the consequence of a reduced proliferation rate of neuroblast. Finally, the authors investigated how Notch signaling intersects with mitochondrial morphology in regulating neuroblast differentiation. Although many findings including those showing the effect of knocking down fusion & fission proteins on the morphology of mitochondria are not novel as they have been shown extensively by others, some data could be a worthwhile contribution to the field of stem cell mitochondrial dynamics. Nevertheless, there are a number of conclusions that require additional support and controls to be rectified.

**Response:** The analysis of the requirement of mitochondrial dynamics in the form of mitochondrial fusion and fission in neural stem cells in *Drosophila* has not been carried out systematically before. Mitochondrial fission has been known to be essential for various stages of stem cell differentiation. In this study we find that mitochondrial fusion and not mitochondrial fission are important for *Drosophila* neural stem cell, neuroblast differentiation. Further loss of mitochondrial fission leads to suppression of differentiation defects seen in mitochondrial fusion mutants. This specificity of requirement for mitochondrial fusion for differentiation of a stem cell lineage raises a number of possibilities for analysis of regulation of mitochondrial fusion by signaling pathways during this differentiation in the very powerful *Drosophila* neuroblast differentiation model system.

Major comments

1. The authors claimed that the phenotypic effects of Opa1-RNAi can be rescued by combining opa1 depletion with the overexpression of a Drp1 dominant negative construct (Drp1sd), which is presumably UAS-Gal4 based (the authors need to specify the nature of the construct in the manuscript somewhere, see below). Theoretically, Drp1sd should promote fusion to counteract Opa1 RNAi induced fission. They also use a similar approach for other RNAi lines (e.g. Marf1 RNAi and notch RNAi) and also combine NotchFL with Opa1 or marf1 RNAi. However, the authors compare these combined genotypes to Drp1SD (or NotchFL) alone, when the correct control would be in combination with another RNAi that has no phenotypic effect (e.g. mCherry RNAi). This is because an increased number of UAS-driven constructs within a genotype can reduce their overall expression by limiting available Gal4. Many of the effects they see when comparing combined genotypes to single genotypes could be explained by such a dosage effect. Therefore, a correct control must be included to draw conclusion based on these experiments.

**Response:** As recommended by the reviewer we have now added controls for expressing the mCherry RNAi along with the Drp1 mutant. Since Drp1^SD^ does not show defects in Dpn or Ase positive cells we also add controls of mRFP in addition to *opa1* and *marf* RNAi in the supplementary. We show that there is no significant difference in mitochondrial area in Drp1^SD^; mCherry RNAi as compared to Drp1^SD^ alone. There is no significant difference between Dpn, Ase and Pros cell numbers in each type II neuroblast lineage in mRFP, Opa1RNAi as compared to Opa1RNAi alone. There is no significant difference between Dpn, Ase and Pros cell numbers in each type II neuroblast lineage in mRFP; *marf* RNAi as compared to *marf* RNAi alone. In summary, addition of mCherry RNAi and mRFP did not change the extent of phenotype of any of the single depletions of mutants used in the study.

What’s more, because Drp1sd is a novel line, the manuscript would benefit from further details about this line in the methods section, such as the vector used to clone the Drp1sd (e.g. UASt or UASp), into which chromosome this construct was inserted? I also wonder why Drp1sd overexpression is used in combination with Opa1/Marf RNAi instead of combining DRP1 RNAi and Opa1/Marf RNAi to achieve double knockdown. Based on Table S1, Drp1 RNAi seems to work well as it gave a similar defect at the organismal level.

**Response:** Mutation at the Drp1S193 locus was created in our previous study [[2]](https://paperpile.com/c/tIJBWg/ydj4). We have added the details of Drp1^SD^ in the materials and methods, it is in the pUASP vector. We have used these mutants instead of the RNAi because the RNAi gave inconsistent phenotypes. When we received a new stock of the RNAi 51483 from Bloomington it did not give any defect in mitochondrial morphology that it originally did and the lack of phenotype has been consistent for 4 shipments of the stock from Bloomington.

We have studied Drp1 mutants extensively in the lab in various different tissues and summarise here and in the revised manuscript the reason for choosing the Drp1^SD^ mutant for further analysis:

1. We show that Drp1^SD^ gives a similar clustered mitochondrial distribution to neuroblast clones of the null allele *drp1*^KG^. Clustered mitochondria with *drp1*^KG^ has been seen earlier in follicle cells, spermatocytes and neurons [[10–13]](https://paperpile.com/c/tIJBWg/PYmj+QWrY+Xwe3+XZAl). Since it was more suitable to perform this analysis by using a dominant negative construct which gives the same phenotype of loss of mitochondrial fission similar to the null allele, we chose to the use the Drp1^SD^ overexpression for analysis of the phenotypes of loss of Drp1 in the type II neuroblast lineage.
2. It is also easier to deplete the function of the Drp1 by adding a dominant negative allele rather than RNAi since Drp1 protein is abundant in several tissues and its function could potentially last several generations of division within a lineage. The null mutants of Drp1, *drp1*^KG^ and the Deficiency for Drp1 locus shows the presence of first instar larvae [[10]](https://paperpile.com/c/tIJBWg/PYmj).
3. Mitochondrial clustering on Drp1 depletion has been seen earlier in mammalian cells [[14,15]](https://paperpile.com/c/tIJBWg/m1Yp+m3v8) and also in Drosophila spermatocytes, neurons, follicle cells and embryos depleted with Drp1 [[2,11–13,16]](https://paperpile.com/c/tIJBWg/XZAl+QWrY+Xwe3+hliT+ydj4). Mitochondrial clusters have been observed to have inter connected mitochondrial tubes in electron microscopy studies in mammalian cells [[15]](https://paperpile.com/c/tIJBWg/m3v8).

2. Despite that the authors used the STED super-resolution imaging to visualize the mitochondrial network in neuroblasts and differentiated cells, the quality of the images is not impressive. It is hard to tell whether those mitochondria in Drp1sd overexpressing cells or notch down-regulating are fused, or simply it is an aggregation of many small mitochondria (e.g. Fig 1B, Figure 3A & Figure 6 etc). A number of images were over-exposed with over-saturated signals, and this will affect the qualifications presented in those figures.

**Response:** STED microscopy improves the resolution of mitochondria stained with ATPb as compared to confocal microscopy in *Drosophila* neuroblasts. We want to study mitochondrial morphology using electron microscopy but do not have this facility in the institute. We have improved our documentation of mitochondria with the STED images. We have used areas of similar sizes across different samples and genotypes containing discrete mitochondria to estimate the mitochondrial area from these cells. We find that there is a significant decrease in mitochondria area in Opa1 and Marf mutant neuroblasts and a significant increase in mitochondrial area in type II neuroblasts mutant for Drp1. In addition Drp1 mutant type II neuroblasts show clustered mitochondrial distribution. Care has been taken to avoid saturation during imaging.

3. Given that Opa1 and Marf knockdown gave a different level of differentiation deficiency, and Opa1 has the additional role in cristae remodelling (which is directly linked to electron chain transport capacity) besides being a mitochondrial fusogen like Marf, it will be great to show the cristae structure by electron microscope after Marf and Opa1 knockdown. However, I understand that performing such additional experiments could be challenging during the pandemic.

**Response:** Opa1 has a role in mitochondrial fusion and in cristae maintenance. The best way of assessing this change is by performing electron microscopy, however we do not have this facility at our institute, we hope that we can add this analysis in the future when travel to another institute is more feasible. Our preliminary analysis shows that similar differentiation defects are also seen in complex V mutants which are important for stabilising cristae. We will indeed pursue a more direct analysis of the role of proteins involved in stabilising cristae in mitochondrial distribution and neuroblast differentiation in a future study.

4. Page 13, 2nd paragraph, the authors state: In summary our results show that mitochondrial fusion controlled by Opa1 and Marf regulate Notch signaling driven differentiation in the Drosophila. I feel the rescue data in Fig 7 are not sufficient to support such a statement. Does Opa1 and Marf knockdown reduce notch signaling? Does notch knockdown lead to decreased Opa1 and Marf expression? In the discussion, the authors stated: our preliminary analysis shows that Opa1 mutant type II NBs show a loss of notch receptors on the plasma membrane and increase in endosomes in the type II NB and the lineage (data not shown). It is an important piece of data to support the link between notch and mitochondrial fusion proteins. Why these data are not shown to further support the statement made in the last paragraph of the discussion, which is “we find a distinct role for mitochondrial fusion in regulation of notch signaling in type II NB differentiation”.

**Response:**

We thank the reviewer for motivating analysis of Notch signaling in the type II neuroblast and their lineage. We have now added this new data as a part of revised figure 6. We find that there is an increase in accumulation of Notch Receptor in the cytoplasm of type II neuroblasts on Opa1 depletion. There is a decrease of Notch Receptor in the lineage in Opa1 depletion. We also estimated the levels of Espl-GFP as a readout for Notch activation in type II neuroblasts and the lineage. We found that there is a significant decrease in Espl-GFP levels in both the neuroblast and the Ase positive cells in the lineage. These data together show that Notch signaling is decreased in Opa1 mutant type II neuroblasts and in the lineage. Both Opa1 and Marf depletion show a defect in proliferation in the type II NB lineage. It is also possible that a defect in the cell cycle, often seen in mitochondrial mutants [[17,18]](https://paperpile.com/c/tIJBWg/R86g+uVlW), is responsible for loss of Notch signaling in Opa1 depleted lineages and loss of Pros positive cells in the Marf depleted type II neuroblast lineages.

The levels of Marf and Opa1 transcription and mitochondrial fusion have been shown to increase in *brat* RNAi. Since the type II neuroblasts are very few in number we have not been able to test the dependence of expression of mitochondrial morphology proteins on Notch signaling. We are in the process of obtaining HA tagged lines for mitochondrial morphology proteins with the endogenous promoter in combination with different mutants to assess the changes in mitochondrial morphology protein expression in neuroblasts and their lineage.

5. At multiple points in the manuscript, the authors used the term “mutants” to refer to RNAi lines or Drp1sd overexpression, which is not correct. Please use a more accurate phrase such as “Opa1 or Marf depleted genotypes” or “combined genotypes”, as relevant.

**Response:** We have made changes in the terminology for the different genotypes used in this study as suggested by the reviewer and updated in the revised manuscript.

Minor comments

1. Some of the panels in supplementary figures could be moved into different figures to aid the flow. Specifically, S2D-G would be better suited in S3 and S4E-G would be better suited as a separate figure.

**Response:**We have changed the panels to suit the flow of the writing in the revised manuscript as suggested and they all appear in order of the writing.

2. Page 5, 1st paragraph, “multiple RNAi lines and mutants” should be replaced by “multiple RNAi lines and overexpression of a dominant-negative Drp1 mutation”.

**Response:** As suggested we have changed the sentence to the following: “We expressed multiple RNAi lines against *opa1*, *marf* and an RNAi line against Drp1 and a dominant negative Drp1 mutation with neuronal Gal4 drivers in different stages of NB differentiation to analyze their effect on lethality and behavior.”

3. Page 7, 3rd paragraph, which Gal4 did the authors use to drive the expression of RNAi and Drp1sd overexpression? Please specify.

**Response:** As suggested, we have added the details of the Gal4 in the corresponding results and legends sections.

4. Page 11, 1st paragraph, “also showed significant increase in PH3” should be replaced by “also showed a significant increase in PH3”.

**Response:** We have added new data here and changed the text for the results as per the new data and also incorporated the changes recommended by the reviewer.

5. In the first paragraph of the section “Mitochondrial fusion induces differentiation on Notch depletion in the type II NB lineage”, please briefly describe relevance of Nintra whilst introducing Notch signaling.

**Response:** We have now moved the Notch intra data to the response letter as we have not addressed whether the Opa1 and Marf depletion gives rise to loss in proliferation or dedifferentiation on the expression of NotchFL. We also agree with the reviewers this is a separate analysis on tumor neuroblasts and hence deserves focussed attention as another study. As suggested by the reviewer we have added a discussion of the tumor neuroblasts from the Bonnay et al 2020 paper in the start of section.

6. Page 11, 2nd paragraph, do the authors intended to say “Notch signaling is activated in type II NBs and suppressed in immature INPs.”?

**Response:** We thank the reviewer for this comment, we have changed this as suggested.

7. Figure 1B – These images would benefit from also showing nuclear staining if the authors already have already done so, because the high background of CD8-GFP makes delineation of the neuroblast difficult to distinguish.

**Response:** Use of nuclear dyes such as DAPI and Hoescht is not recommended for the STED imaging as it might have a negative effect on image quality. Hence we did not perform nuclear staining in the mitochondrial imaging using STED.

8. Figure 1C - A limitation of this quantification of mitochondrial area and number is that these measurements were done on a single plane. As it is difficult to overcome this limitation, it would be useful to have this clarified in the figure legend.

**Response:** As suggested we have added this description to the results, legend and materials and methods.

9. The final paragraph of the section “Mitochondrial fusion is required for differentiation in the type II NB lineage” would be more suitable for the discussion, so should be moved or shortened.

**Response:** We have changed this to “Mitochondrial fusion by Drp1 depletion induces differentiation in Notch depleted type II NB lineages”

10. Figure 3D – The same control images has been used for Figure 2 and 3. It therefore may be more appropriate to group Figures 2 and 3 rather than repeat data.

**Response:** *pnt*-Gal4, UAS-mCD8-GFP and mCherry RNAi are used as a control in Figure 2 and Figure 3 and S4 has many controls for comparison with double mutants:

*pnt*-Gal4, UAS-mCD8-GFP

Drp1^SD^, mCherry RNAi

*opa1* RNAi

mRFP; *opa1* RNAi

*marf* RNAi

mRFP; *marf* RNAi

11. Figure 4A – What the numbers on the panels represent has not been stated or, if stated, should be made clear.

**Response:** In the Figure 4A, we have analyzed mitochondrial membrane potential across different genotypes. Numbers on panels represent the percentage of NBs (out of total analyzed NBs) which showed membrane potential similar to the represented images. This description is updated in the legend part of the figure.

12. Figure 4B and associated text - Please expand on how neighboring cells were selected and identified for comparison (also for S4B and S4D).

**Response:** We have computed the intensity of DHE in the type II neuroblasts expressing mCD8-GFP and expressed this as a ratio to the neighboring unmarked neuroblasts in the same optical plane. This information has been updated in the materials and methods.

13. Figure 7G – There is inconsistency to other figures because the NB number has not been normalized per hemisphere. Is there a reason for this?

**Response:** Neuroblast numbers in all figures have been counted per hemisphere and this information has been updated in the legends and the materials and methods.

Bibliography

1.	[Mao L, Li N, Guo Y, Xu X, Gao L, Xu Y, et al. AMPK phosphorylates GBF1 for mitotic Golgi disassembly. J Cell Sci. 2013;126: 1498–1505.](http://paperpile.com/b/tIJBWg/gyfT)

2.	[Chowdhary S, Madan S, Tomer D, Mavrakis M, Rikhy R. Mitochondrial morphology and activity regulate furrow ingression and contractile ring dynamics in Drosophila cellularization. Mol Biol Cell. 2020; mbcE20030177.](http://paperpile.com/b/tIJBWg/ydj4)

3.	[Chowdhary S, Tomer D, Dubal D, Sambre D, Rikhy R. Analysis of mitochondrial organization and function in the Drosophila blastoderm embryo. Sci Rep. 2017;7: 5502.](http://paperpile.com/b/tIJBWg/62A1)

4.	[Bonnay F, Veloso A, Steinmann V, Köcher T, Abdusselamoglu MD, Bajaj S, et al. Oxidative Metabolism Drives Immortalization of Neural Stem Cells during Tumorigenesis. Cell. 2020;182: 1490–1507.e19.](http://paperpile.com/b/tIJBWg/0JZM)

5.	[Li X, Xie Y, Zhu S. Notch maintains Drosophila type II neuroblasts by suppressing expression of the Fez transcription factor Earmuff. Development. 2016;143: 2511–2521.](http://paperpile.com/b/tIJBWg/YHkN)

6.	[Deng Q, Guo T, Zhou X, Xi Y, Yang X, Ge W. Cross-Talk Between Mitochondrial Fusion and the Hippo Pathway in Controlling Cell Proliferation During Drosophila Development. Genetics. 2016;203: 1777–1788.](http://paperpile.com/b/tIJBWg/XX11)

7.	[Wong KKL, Liao JZ, Shih CRY, Harden N, Verheyen EM. Hyperpolarized mitochondria accumulate in Hipk-overexpressing cells to drive tumor-like growth. J Cell Sci. 2020;133. doi:](http://paperpile.com/b/tIJBWg/7J2z)[10.1242/jcs.250944](http://dx.doi.org/10.1242/jcs.250944)

8.	[Deng H, Dodson MW, Huang H, Guo M. The Parkinson’s disease genes pink1 and parkin promote mitochondrial fission and/or inhibit fusion in Drosophila. Proc Natl Acad Sci U S A. 2008;105: 14503–14508.](http://paperpile.com/b/tIJBWg/4TrL)

9.	[Dorn GW 2nd, Clark CF, Eschenbacher WH, Kang M-Y, Engelhard JT, Warner SJ, et al. MARF and Opa1 control mitochondrial and cardiac function in Drosophila. Circ Res. 2011;108: 12–17.](http://paperpile.com/b/tIJBWg/lOXD)

10.	[Rikhy R, Kamat S, Ramagiri S, Sriram V, Krishnan KS. Mutations in dynamin-related protein result in gross changes in mitochondrial morphology and affect synaptic vesicle recycling at the Drosophila neuromuscular junction. Genes Brain Behav. 2007;6: 42–53.](http://paperpile.com/b/tIJBWg/PYmj)

11.	[Mitra K, Rikhy R, Lilly M, Lippincott-Schwartz J. DRP1-dependent mitochondrial fission initiates follicle cell differentiation during Drosophila oogenesis. J Cell Biol. 2012;197: 487–497.](http://paperpile.com/b/tIJBWg/QWrY)

12.	[Tomer D, Chippalkatti R, Mitra K, Rikhy R. ERK regulates mitochondrial membrane potential in fission deficient Drosophila follicle cells during differentiation. Dev Biol. 2018;434: 48–62.](http://paperpile.com/b/tIJBWg/XZAl)

13.	[Aldridge AC, Benson LP, Siegenthaler MM, Whigham BT, Stowers RS, Hales KG. Roles for Drp1, a dynamin-related protein, and milton, a kinesin-associated protein, in mitochondrial segregation, unfurling and elongation during Drosophila spermatogenesis. Fly . 2007;1: 38–46.](http://paperpile.com/b/tIJBWg/Xwe3)

14.	[Smirnova E, Griparic L, Shurland DL, van der Bliek AM. Dynamin-related protein Drp1 is required for mitochondrial division in mammalian cells. Mol Biol Cell. 2001;12: 2245–2256.](http://paperpile.com/b/tIJBWg/m1Yp)

15.	[Smirnova E, Shurland DL, Ryazantsev SN, van der Bliek AM. A human dynamin-related protein controls the distribution of mitochondria. J Cell Biol. 1998;143: 351–358.](http://paperpile.com/b/tIJBWg/m3v8)

16.	[Insolera R, Lőrincz P, Wishnie AJ, Juhász G, Collins CA. Mitochondrial fission, integrity and completion of mitophagy require separable functions of Vps13D in Drosophila neurons. bioRxiv. 2020. p. 2020.01.21.914523. doi:](http://paperpile.com/b/tIJBWg/hliT)[10.1101/2020.01.21.914523](http://dx.doi.org/10.1101/2020.01.21.914523)

17.	[Chen H, Chan DC. Mitochondrial Dynamics in Regulating the Unique Phenotypes of Cancer and Stem Cells. Cell Metab. 2017;26: 39–48.](http://paperpile.com/b/tIJBWg/R86g)

18.	[Parker D, Iyer A, Shah S, Moran A, Hjelmeland A, Basu MK, et al. A novel mitochondrial pool of Cyclin E, regulated by Drp1, is linked to cell density dependent cell proliferation. Journal of Cell Science. 2015. doi:](http://paperpile.com/b/tIJBWg/uVlW)[10.1242/jcs.172429](http://dx.doi.org/10.1242/jcs.172429)
