# Supplementary material for: Mitochondrial fusion regulates proliferation and differentiation in the type II neuroblast lineage in Drosophila
Source: PLoS Genet. 2022 Feb 14;18(2):e1010055. doi: 10.1371/journal.pgen.1010055 (PMC8880953; doi:10.1371/journal.pgen.1010055)
Supplement: Attachment — Submitted filename: Dubal et al Response to Reviewers.docx [file pgen.1010055.s028.docx]

The pointwise response to the reviewer’s questions is given below. The response to the reviewers is also uploaded as an attachment.

Reviewer's Responses to Questions

**Comments to the Authors:**

**Summary of revisions carried out in response to comments by Reviewer 1:**

We thank the reviewers for giving us detailed comments and working with us to revise the manuscript. We have revised the manuscript further to address the comments of the reviewers. We summarise the revisions performed in this manuscript below:

1. We obtained the Dpn and Ase antibodies in different animals from the Jan lab and estimated the numbers of GMCs (Dpn- and Ase+ cells) as defined by the literature. We show that there is a decrease in GMCs on Opa1 depletion and an increase in GMCs on Marf depletion (Figure 2,3,7,S3).
2. We show mitochondrial morphology analysis in type II neuroblasts, mature INPs and muscle cells (Figure 1,3,6,S2,S10).
3. We show that mitotic neuroblasts decrease on Opa1 depletion and this phenotype is reversed on mitochondrial fusion in Drp1 mutant cells (Figure 5).
4. We have compared the numbers of Elav positive and brightly stained Pros positive cells in each lineage in the response letter. They are comparable in number. Also, clonal analysis of Marf RNAi mutant lines shows a decrease in numbers of brightly stained Pros positive cells similar to pnt-Gal4, Marf RNAi (Response letter Figure 1).
5. We have analyzed the activation of Notch signaling in the type II neuroblast lineages (Figure 6).

We find that there is a decrease in type II neuroblast proliferation and loss of Notch signaling on depletion of Opa1. This defect is not seen on additional expression of mutant mitochondrial fission protein Drp1.

**Reviewer #1**: The manuscript has improved to some extent and authors have address some of my concerns. However, some critical concerns I had before still have not been addressed. Some of their conclusions are still not well supported by their data and some data could be even mis-leading. Given that there are still so many concerns/problems after revision, I would not support publication of the manuscript in PLoS Genetics.

Here are some of the major concerns that still remain to be addressed (most of these concerns are the ones I mentioned previously).

1. The authors showed that Opa1 knockdown led to a reduction in the number of Dpn+ cells, Ase+ cells, and Pros [high] cells. By co-staining of pH3, they further showed that mitotic rate of mINPs (Dpn+ cells) was reduced. Based on these data, the author concluded that the reduction of Dpn+ cells was because of the reduced mitotic rate of mINPs. However, if mINPs have a reduced mitotic rate, there should be an increase in the number of mINPs (as they argued that reduction in the mitotic rate likely accounts for the increased number of GMCs in Marf knockdown lineages) unless the number of cell cycles that mINPs will undergo is reduced and mINPs terminate their self-renewal prematurely. They have to look at the mitotic rate of type II NBs upon knockdown of Opa1 (and Marf knockdown as well) as I suggested previously. Reduction in the mitotic rate of type II NBs certainly will also lead to a reduction in the number of mINPs and more likely is the real reason.

**Response:**

As recommended by the reviewer we have added the estimation of the numbers of the mitotic type II neuroblasts. We find that the mitotic rate of neuroblasts is decreased in Opa1 depleted type II neuroblasts. The mitotic rate of mINPs in the type II neuroblast lineage is also decreased. There is a decrease in numbers of mINPs, GMCs and neurons in each type II neuroblast lineage depleted of Opa1 RNAi.

The division rate of the type II neuroblasts does not change upon Marf depletion. The numbers of mINPs do not change significantly on Marf depletion. However, their division rate is higher than controls. The division of mINP leads to self renewal, increase in mitotic rate of mINPs will lead to increase in the numbers of GMCs without changing the mINPs. The GMCs also divide less than controls thereby causing a decrease in the number of neurons. The increase in the mitotic rate of mINPs and decrease in the rate of division of GMCs leads to their accumulation.

We also tested the division rate of type I and type II NBs using *worniu* Gal4 and found that the rate of division was decreased in Opa1 RNAi expression and not in Marf RNAi expression.

2. The authors showed that Marf knockdown resulted in an increased mitotic rate of Dpn+ cells (mINPs), an increased number of Ase+ cells, and a reduction in the number of Pros[high] cells, without changes in the number of mINPs. They concluded that the number of GMCs was increased but GMCs failed to produce neurons or produced much fewer neurons. They offered two different explanations for the increase in the number of GMCs and reduction in the number of Pros[high] cells: increased proliferation of mINPs and reduced proliferation of GMCs in the results and discussion parts. There are many problems here. First, if mINPs divide more quickly than usual, then the number of Dpn+ mINPs should be reduced unless mINPs can undergo more cell cycles than wild type mINPs. They should provide data to show if the number of cells cycles of mINPs is changed or not (e.g. by examining the number of neurons generated in an INP clone).

**Response:**

As suggested by the reviewer, we have added new data for numbers of mitotic GMCs in each type II neuroblast lineage on Marf depletion. We find that the mINPs do not increase significantly on Marf RNAi expression. GMCs increase in numbers as compared to controls. Brightly stained Pros positive cells labeling neurons however decrease. There is an increase in the proliferation of mINPs. Our new data shows reduction of proliferation of GMCs and this leads to the reduction of the number of neurons. The increase in GMCs therefore occurs due to the combined effect of increased proliferation of mINPs and decreased proliferation of GMCs.

Second, if they think that the number of GMCs was increased after Marf knockdown, they need to do Ase and Pros co-staining rather than Ase staining alone to un-ambiguously identify GMCs for quantifying the number of GMCs.

**Response:**

As suggested by the reviewers we have added new data for quantification of GMCs by staining with Dpn and Ase together (Dpn- and Ase+ cells are GMCs in the type II neuroblast lineage). We find that there is an increase in GMCs in Marf RNAi and a decrease in GMCs in Opa1 RNAi. These phenotypes are suppressed on the additional expression of Drp1SD mutants.

Third, they need to examine the mitotic rate of GMCs specifically by co-staining Ase, Pros, and pH3, if they suspect that GMCs do not divide as actively as usual after knockdown of Marf. It seems odd that knockdown of Marf would increase the proliferation of mINPs but decrease the proliferation of GMCs. How could knockdown of Marf have totally different effects in INPs and GMCs in terms of cell proliferation?

**Response:**

As suggested by the reviewer we have analysed the mitotic rate of GMCs by staining with pH3, Ase and Pros in type II neuroblast lineages depleted of Marf. We find that there is a decrease in the proliferation of GMCs in Marf RNAi depleted type II neuroblast lineages. These data suggest that the GMCs formed are defective and not able to proliferate to form differentiated cells.

As pointed out by the reviewer, there is a difference in cell cycle defects in mINPs and GMCs in Marf depleted type II neuroblast lineages. At this point, we conclude that the GMCs produced by the highly mitotic mINPs are defective and are unable to divide and produce differentiated neurons.

3. Quantifying the number of neurons in type II NB lineages by counting the number of Pros[high] cells in type II NB lineages labelled with mCD8-GFP driven by pnt-GAL4 is not a proper way for quantifying the number of neurons and the results could be misleading. The reason is because pnt-Gal4 is only active in type II NBs and imINPs and mCD8-GFP expression in the progeny decreases gradually and dose not label all the cells produced in individual type II NB lineages. The expression of mCD8-GFP driven by pnt-GAL4 does not tell where is the boundary of the lineage. They have to label the lineages with mCD8-GFP driven by either elav-GAL4 or tub-GAL4 and compare the number of neurons in MARCM clones or flipout clones.

**Response**:

Quantification of neurons in type II NB lineages is carried out by counting the number of Pros (high) cells, which are present at the bottom in type II NB lineage. As pointed out by the reviewer that *pnt*-Gal4 driven expression of mCD8-GFP decreases gradually in the progeny. We have stained brains with anti-GFP antibodies, which could label cells even with the low GFP signal. During the analysis of neuronal numbers in the ImageJ software, we increase the brightness of the GFP channel using the B & C (brightness and contrast) tool to visualize the boundary of each lineage. Wherever determination of the boundary with GFP channel is not possible, we omit that lineage from the analysis. Further to confirm this observation, we stained brains with anti-Elav antibodies to mark neuronal population in type II NB lineage and performed similar quantification. There was no significant difference in Elav+ and Pros(high) cells in mCherry RNAi control (response letter figure 1A,C). As suggested, we also performed MARCM based clonal experiments where the entire clone is labeled uniformly with *tubulin*-Gal4, mCD8-GFP to show a decrease in Pros positive cells on Marf depletion (response letter figure B,C).

We did not get mINPs clones in this study and are interested to work on this aspect to analyze the cell cycle in Marf RNAi clones and other mutants that show the same defect in a future study.

Response Letter Figure 1:

A. *pnt*-Gal4, UAS-mCD8-GFP expressing brains from third instar larvae are stained with Elav and Pros. Brightly stained Pros expressing neurons and Elav positive neurons are comparable in numbers. There is a loss of bright Pros labelled neurons in the Marf RNAi as shown in Figure 2 in the revised manuscript.
B. Clones generated by using the MARCM system show brightly stained Pros positive neurons at the base in control clones and are decreased in Marf RNAi expressing clones.
C. Graph showing a comparison between neuron numbers in each type II neuroblast lineage labelled with Pros and Elav. There is a decrease in Pros positive neurons in Marf RNAi expressed with pnt-Gal4. The numbers of Pros Bright neurons in the MARCM clone containing Marf RNAi is comparable to that seen with Marf RNAi expressed with pnt-Gal4.

The n value indicated in the image is the number of independent brains and each dot represents counts from a single lineage. Graphs show mean + sd. Comparative analysis was done by using unpaired t-test. ns- non significant, **- p<0.01, ***- p<0.001

4. Throughout the entire paper, the mitochondria morphology is examined in type II NBs, however, the proliferation/differentiation defects were mostly observed in INPs/GMCS/neurons. Are the defects in mitochondrial morphology observed in type II NBs resulting from Opa1/Marf/Drp1 knockdown/inhibition or Notch manipulations really responsible for the proliferation/differentiation defects of INPs/GMCs? Or actually do these manipulations cause similar mitochondrial morphological defects in INPs/GMCs, which in turn impact their proliferation/differentiation?

The authors should examine the behavior of type II NBs and mitochondrial morphology in INPs/GMCS after Opa1/Marf/Drp1 knockdown/inhibition or Notch knockdown/overexpression.

**Response:**

As recommended by the reviewer, we have estimated the rate of mitosis in type II neuroblasts. We find that the type II NB has decreased in proliferation on Opa1 depletion. We have also estimated the rate of division of neuroblasts in the entire brain and found a decrease in the numbers of mitotic neuroblasts on Opa1 depletion.

We have also estimated the proliferation in the GMCs and find that this is decreased in Marf depleted lineages.

We have added the mitochondrial morphology analysis in the mINPs of the type II neuroblast lineage. We find that Drp1SD expressing type II neuroblast lineages contained clustered mitochondrial organization in mINPs. Mitochondria are dispersed in mINPs expressing Opa1 RNAi and Marf RNAi similar to controls. Our qualitative analysis shows that mitochondria appear more dispersed in Opa1 and Marf RNAi expressing mINPs as compared to controls. mINPs expressing double knockdown show clustered mitochondria like Drp1SD mutants. These data are a part revised Figures 1 and 3.

Mitochondria are somewhat clustered on Notch overexpression and are fragmented in Notch and Su(H) RNAi. This new data is a part of revised Figure S10.

We summarise that a semi-quantitative analysis for mitochondrial area is possible in the type II neuroblast in discrete locations and only qualitative visual analysis is possible in the mINPs since they are much smaller cells present in deeper sections and in different orientations in the lineage.

5. In terms of mitochondrial morphological defects described in the manuscript, only thing obvious and convincing to me is that expressing Drp1SD leads to aggression of mitochondria. I am not sure if anyone can see the mitochondrial morphological defects described for Opt1/Marf/Notch or overexpressing NotchFL/Nintra without looking at the quantification data. Furthermore, in Drp1SD expressing NBs, mitochondria all aggregate together, how could they measure the area of individual mitochondria. If they measured the region where they could see individual mitochondria as they mentioned, then obviously the data are not representative and does not truly reflect the phenotype observed in Drp1SD expressing NBs.

**Response:**

As recommended by the reviewer, we have included a qualitative analysis of mitochondrial morphology by classifying them into intermediate, clustered and fragmented in all the genotypes in type II neuroblasts and mature INPs and added the percentage of maximum phenotype to the legend. This is added to revised Figure 1, 3, 6, S10.

We can estimate the relative mitochondrial distribution and not mitochondrial morphology even though we attempted STED super-resolution microscopy, which has a higher resolution than confocal microscopy. The mitochondrial area can only be estimated in regions where mitochondria are present discretely using confocal and STED microscopy. The most striking phenotype is mitochondrial clustering in the Drp1 mutant expressing type II neuroblasts.

We have now included a comparison of changes from muscle cells where discrete mitochondria are visible in revised Figures S2 and S10. We find that mitochondria are larger in Drp1SD expressing muscle cells and smaller in the cells expressing Opa1 and Marf RNAi compared to controls. As we mentioned in the previous response letter, electron microscopy would be ideal to confirm this; however, we do not have access to transmission electron microscopy, but this is our focus. We will study mitochondrial architecture using transmission electron microscopy in the future.

We have also added an analysis of mitochondrial morphology in the lineage in Notch overexpression and RNAi lineages in mINPs. We find that mitochondrial morphology is somewhat clustered on Notch overexpression and dispersed in Notch RNAi expressing type II neuroblast lineages. This data is a part of revised Figure S10.

We also request the reviewers to note that mitochondrial morphology changes in transmission electron microscopy images in these RNAis have been carried out in previous studies [[1–5]](https://paperpile.com/c/bRdn0h/VrnW+mMyq+qKsY+qz66+tc38).

6. As I mentioned previously, the data presented in this manuscript are not consistent with a published paper (Bonnay F, et al, Cell 2020, 182, 1490) in terms of the number of mINPs in Marf and Opa1 knockdown type II NB lineages. The authors should test the same VDRC RNAi lines used by Bonnay F et all to see if they can reproduce the results. They should also discuss the discrepancy of the results in the discussion part.

**Response:**

As per the suggestion of reviewer 1, we tested the VDRC lines for our revised manuscript. We found that similar to the previous 2 RNAi lines that we had tested for Opa1 depletion; the VDRC line also showed the same reduction of Dpn positive cells in the type II lineage. GMCs were also reduced on Opa1 depletion in the VDRC line. We have added this data to the revised Figure S2. We also find an increase in GMCs on Marf depletion in the VDRC line. We have mentioned that there is a discrepancy between our results and Bonnay et al 2020. We cannot explain how this difference is seen between our analysis and Bonnay et al 2020. However, we request the reviewer to note that we get the same decrease in mINPs in 3 different RNAi lines against Opa1.

7. The authors suggest that Notch signaling regulates mitochondrial fission/fusion by activating the expression of Opa1, which in turn regulates Notch signaling by promoting translocation of NICD from the cytoplasm to the nucleus. So there is a positive feedback loop of Notch signaling and Opa1. However, I don’t find that their data are convincing enough to support that Opa1 regulates Notch signaling by regulating the nuclear import of NICD. The biggest concern I have is that E(Spl)-GFP [by the way, it should read E(Spl)m-GFP as there are multiple different E(Spl) genes] signal is reduced in type II NBs when prospoero-GAL4 drives the expression of UAS-opa1 RNAi (Fig6E-G). Prospero-GAL4 is not expressed in type II NBs at all. It is only expressed in INPs and GMCs. Therefore, Opa1 is not knocked down in type II NBs at all when UAS-opa1 RNAi is driven by prospero-GAL4. How could E(Spl)m -GFP expression in type II NBs be affected in this case? By the way, comparing the E(Spl)m-GFP expression levels by comparing the signal/noise ratio certainly is not the best way for quantifying the E(Spl)m-GFP expression.

**Response:**

We have now added an analysis of E(spl)mγ-GFP in the type II neuroblast lineage by expressing the RNAi and mutants using pointed Gal4 in the revised manuscript. We find that there is similar activation of E(spl)mγ-GFP in the type II neuroblasts in Opa1, Marf and Drp1 knockdown as compared to controls. There is a decrease in E(spl)mγ-GFP expression in the lineage on Opa1 depletion. There is feedback between Opa1 and Notch Receptor trafficking and signaling in the type II neuroblast lineage. Notch signaling activation as shown by E(spl)mγ-GFP is not affected significantly in Opa1 RNAi expressing type II neuroblasts. These data are a part of revised Figure 6H. Based on these results of feedback between Opa1 and Notch signaling, we have now revised the summary schematic in Figure 8 to document the different phenotypes seen on Opa1 depletion.

We also find a defect in Notch receptor distribution using the NICD (Figure 6) and NECD (Response letter figure 2) antibodies in the type II neuroblast and in the lineage on Opa1 depletion. Defects in the cell division and Notch trafficking are likely to give rise to loss of mINPs and GMCs in the lineage on Opa1 depletion.

Furthermore, I am not totally convinced that NICD staining could be used to assess the activation of Notch receptors in this case. The NICD antibody recognize both the uncleaved Notch as well as cleaved NICD. And I do not see clear NICD signal in the nucleus. In fact, in their discussion part, the authors state that “our observations show that Opa1 depleted type II NBs show a loss of Notch receptors on the plasma membrane and increase in endosomes in the type II NB and in the cells of the lineage”, which is not shown in their figures at all. In Fig 6C-D, they only compared the ratio of cyto/nuclear of NICD staining intensity. Therefore, more convincing data are needed to further support that Opa1 regulates Notch signaling. It is hard to imagine that mitochondria proteins like Opa1 would regulates the translocation of NICD from the cytoplasm to the nucleus. The authors did not provide any reasonable explanation either in the discussion part. If they cannot provide more convincing data, the model proposed in Fig 8 should also be modified.

**Response:**

We present data with NICD (Figure 6) and NECD (Response letter figure 2) in the type II neuroblast and in the lineage for Opa1 knockdowns. The loss of Notch distribution in the type II NB does not lead to a defect in Notch signaling activation as seen by E(spl)mγ-GFP in the NB. However, the Notch activation signal is lost in the Opa1 depleted type II neuroblast lineage cells. We have revised the description of the quantification to state that NICD fluorescence accumulates in the cytoplasm. In the supporting Response letter figure 2, we also show an accumulation of NECD in the cytoplasm in the form of punctae.

Dubal et al 2021 Response letter Figure 2

Response Letter Figure 2: Knockdown of Opa1 in type II neuroblast lineage causes increased NECD punctae in the neuroblast and differentiated cells.

A: Representative confocal images of type II lineages stained with CD8-GFP (green) showing increased NECD (red) punctae in neuroblast (grey dotted line, yellow arrows) and in differentiated cells in the lineage (yellow dotted line, white arrows) depleted for Opa1. Control (15 NBs, 4 Brains), *opa1* RNAi (16,5), *marf* RNAi (14,5).

B: Quantification of NECD punctae in type II neuroblasts. Control (15 Neuaroblasts, 4 Brains), *opa1* RNAi (16,5), *marf* RNAi (14,5).

C: Quantification of NECD punctae (each punctae area ≥ 0.1uM^2^) in differentiated lineage. Control (13 type II NB lineages, 4 Brains), *opa1* RNAi (15,5), *marf* RNAi (14,5).

Graphs show mean + sd. Comparative analysis was done by using unpaired t-test. ns- non significant, *- p<0.05, ***- p<0.001

8. In lines 375-277, the authored stated that “We also observed a change in the mitochondrial morphology …… (Figures S8A-B)”. However, in that figure, they only show the stainining of Dpn and Ase and wild type and NotchFL overexpressing lineages. Also, in this figure, I would expect to see ectopic type II Nbs in NotchFL overexpressing lineages, but the lineages shown in the figure look pretty normal and did not have any extra type II NB lineages. Why?

**Response:**

We have revised the figure callout to represent the figure correctly. We have now included a complete lobe area in the revised manuscript to show Dpn in the Notch FL. We observed ectopic NBs in NotchFL. The increased numbers of NBs in NotchFL were partially rescued by Opa1 RNAi and Marf RNAi expression. This revised data is now added in the manuscript in figure S7A-B.

9. In my previous comments (#12), I suggested to examine the expression of Ase in in Drp1SD Notch RNAi type II NBs to further elucidate how Drp1SD expression could rescue the loss of mINPs in Notch RNAi lineages. However, they still did not do it. In response to my comment, they stated that “2. Ase is not present in the type II neuroblast lineages at the third instar larval stage in Drp1SD; notch RNAi” and cited again Figure S8. However, Fig S8 is about NotchFL overexpression, not notch RNAi. So they cited the same figure wrongly at least twice (see above #8). They also argued that “3. We have Dpn and Ase antibodies in the same animal and hense we could not check for loss of conversion of type I by dissecting brains at earlier time points”. This does not sound a reasonable excuse for not examining the Ase expression in Drp1SD, notch RNAi type II NBs. First, the check the expression of Ase in Drp1SD, notch RNAi type II NBs, it does not require co-staining of Dpn and Ase. Ase staining alone is sufficient. mCD8-GFP driven by pnt-GAL4 would allow for identification of type II NBs. Second, the ectopic Ase expression (thus the conversion of type II NBs into type I NBs can be observed in late larval stages when Notch is knocked down according to published papers.

Response:

We have provided this data in revised figure 7A. We find that Ase is not expressed in Drp1SD, NotchRNAi NBs.

We have also performed new experiments to estimate the numbers of mINPs and GMCs in each type II lineage in all the genotypes. This data is a part of the revised figure 7A,D-E

10. Related with the rescue of Notch RNAi phenotypes by Drp1SD, the authors showed that Drp1SD could rescued the number of mINPs and Ase+ cells but not Pros[high] cells. That does not seem to make sense. The authors should explain how this could happen.

**Response:**

We observed that Dpn+ mature INPs were rescued by co-depletion of Drp1 in the Notch depletion background. However, we only observed partial rescue for Ase+ cells in the type II lineage. Asense is present in both mature INPs and GMCs. This led us to speculate that partial rescue might result from increased mature INPs and not GMCs.

To evaluate this observation further, we have now performed co-staining of Ase and Dpn in *notch* RNAi and Drp1SD;*notch* RNAi genotypes along with the appropriate controls. Mature INPs are positive for both Ase and Dpn, while GMCs express Ase and not Dpn. We see that the Dpn positive mature INPs are increased in numbers in Drp1SD;*notch* RNAi double mutants as compared to Notch depletion alone but do not reach control levels. We did not see an increase in Ase+ Dpn- GMCs on co-depletion of Notch and Drp1. These results show that mINPs are being produced at increased numbers but they are defective and do not produce GMCs at the appropriate level. The bright Pros cells also remain decreased in these combinations. We have added this data to the revised manuscript (Figure 7).

Other minor concerns:

11. In lines 419 – 421, they stated that “the loss of Dpn positive cells in notch RNAi… was suppressed completely … (Fig 7A, E)”, but the bar graph in Fig7E still show statistically significant difference between +/+ and Drp1SD; notch RNAi. So the suppression is not complete. The statement should be modified.

Response: We have rectified this observation in the revised manuscript, The numbers of Dpn positive cells increases in Drp1SD, Notch RNAi as compared to Notch RNAi but do not become similar to controls.

12. In lines 542 – 545, the authors stated that “Drp1 mutant …. that Drp1 is dispensable for NB formation….”. It is an overstatement because the expression of Drp1SD is driven by pnt-GAL4, which is only expressed after type II NBs are formed. Therefore, they cannot conclude that Dpr1 is dispensable for NB formation.

Response: We have changed this statement to the following: These data suggest that Drp1 activity is not required for type II NB differentiation while Opa1 and Marf depletions impairs differentiation in the type II NB lineage.

13. Line 627: dilution of the Ase antibody should be indicated.

Response: We have used 1:2000 dilution for the rat-anti-Ase antibody and the same is added in the materials and method section in the revised manuscript as per the suggestion.

14. For estimating the number of Ase+ neurons, they used the average area of Pros+ nuclei of 5 GMCs (lines 759-765). However, the size of GMCs may not necessary be the same as neurons. They should use the average area of Pros+ nuclei of neurons instead of GMCs.

Response: This has been corrected in the revised manuscript to estimating the average area of Pros positive nuclei.

15. In fig6C opa1 RNAi, what do the yellow arrows point to? It is not mentioned in the figure legends.

Response: The yellow arrows point to the lineage cells which are positive for both deadpan and pH3. We have added this information to the legends.

16. Fig 7E, Y-axis, Dpn+ve should be changed to Dpn+

Response: The Y axis has been changed to represent the names of the cell types in the revised figures.

**Summary of revisions carried out in response to comments by Reviewer 2**

We thank the reviewer for giving us comments to revise the manuscript. We summarise the revisions performed in this manuscript below:

1. We obtained the Dpn and Ase antibodies in different animals from the Jan lab and estimated the numbers of GMCs (Dpn- and Ase+ cells) as defined by the literature. We show that there is a decrease in GMCs on Opa1 depletion and an increase in GMCs on Marf depletion (Figure 2,3,7,S3).
2. We show mitochondrial morphology analysis in type II neuroblasts, mature INPs and muscle cells (Figure 1,3, 6, S2, S10).
3. We show that mitotic neuroblasts decrease in Opa1 depleted type II neuroblasts, and this phenotype is reversed on mitochondrial fusion in Drp1 mutant cells (Figure 5).

**Reviewer #2:** The authors have addressed some of my concerns from the previous version of the ms, and in general, the data quality has improved. I however still have several reservations.

I have still a problem with the data shown in Figure 1, the authors claim that there is fragmentation of mitochondria, and indeed in the particular ROI they have chosen, the quantification is reflective of their observation. However, it is not clear to me how this box is chosen in the top three panels of Figure 1B. To make this point more convincing, the authors should choose multiple ROIs in the same section and perform the analysis, as a minimum. Similar point applies to Figure 3A, the claim that the phenotype is to do with fragmentation of the mitochondria is still not very convincing. In Figure 3, seems like the more striking and perhaps easier to quantify phenotype is the aggregation of the mitochondria to one side of the neuroblast, and maybe the total amount of mitochondria is increased in Drp1SD and similarly in combination with opaRNAi and MarfRNAi. Is there an explanation as to why the fusion causes aggregation of the mitochondria to one side of the NB?

**Response:**

In the revised manuscript, we have added images of mitochondrial morphology in muscle cells in various genotypes to highlight the change in area in mitochondria. The image shows larger mitochondria in Drp1 mutants and smaller mitochondria in Marf and Opa1 RNAi expressing muscle cells. This change in the area has been noted earlier in muscle cells by confocal microscopy and also transmission electron microscopy in the same lines [[4,6]](https://paperpile.com/c/bRdn0h/72uJ+qz66).

We have classified mitochondrial morphology into clustered, fragmented and intermediate and obtained percentage phenotype in the type II neuroblasts and mature INPs. We have added this data for all the genotypes in the revised manuscript.

It is indeed difficult to resolve mitochondria in regions where they are crowded. Therefore we have chosen regions where mitochondria are present discretely. We have done this analysis from multiple cells across different brains as suggested and documented the numbers used in the legends. We have also added the n value for the observation of mitochondrial aggregation in mINPs in the revised manuscript. As suggested we have mentioned an analysis of aggregation of mitochondria as a percentage also.

The density of mitochondria may change in the morphology mutants, this is especially apparent in the mature INPs on depletion of Marf and Opa1, we have discussed this possibility in the results and discussion section.

In Figure 2H, as the authors are unable to show Dpn and Ase together due to technical reasons, it is a claim too far to say that it is definitely mature INP to GMC transition that is impacted in MarfRNAi. Furthermore, in the summary diagram, if Dpn/Ase double positive cells could not be ascertained, I don’t see how it can be summarised as such in the diagram.

**Response:**

We have now performed the Dpn and Ase staining together with new antibodies obtained from the Jan lab raised in different animals to analyze GMC population with more clarity. We quantified Dpn- Ase+ cells in type II lineage across different genotypes. We found reduced GMCs in Opa1 RNAi while GMCs were increased in Marf RNAi, further confirming the previous observations. Co-depletion of Drp1 rescues GMC number in both Opa1 and Marf depletions. We have added this data to the revised manuscript. The summary figure in Figure 2H is added in accordance with the new data.

**Summary of revisions carried out in response to comments by Reviewer 3**

We thank the reviewer for giving us comments to revise the manuscript. We summarise the revisions performed in this manuscript below:

1. We obtained the Dpn and Ase antibodies in different animals from the Jan lab and estimated the numbers of GMCs (Dpn- and Ase+ cells) as defined by the literature. We show that there is a decrease in GMCs on Opa1 depletion and an increase in GMCs on Marf depletion (Figure 2,3,7,S3).
2. We show mitochondrial morphology analysis in type II neuroblasts, mature INPs and muscle cells (Figure 1,3, 6, S2, S10).
3. We show that mitotic neuroblasts decrease in Opa1 depleted type II neuroblasts and this phenotype is reversed on mitochondrial fusion in Drp1 mutant cells (Figure 5).

Reviewer #3: The authors have put in a commendable effort to improve the manuscript. They have considered many of the reviewer’s comments and implemented most in some way. In particular, changes to the manuscript writing, figure legends and explanations of methodology and stocks has improved understanding of their presented results. The additional results obtained showing reduced Notch signaling upon Opa1 knockdown are very interesting and provides additional evidence for a link between Notch signaling and mitochondrial morphology in differentiation that will be exciting to follow up.

**Response:**

We thank the reviewer for encouraging comments on the revision. We have revised the manuscript further to incorporate suggestions from the reviewers.

However, the authors have still failed to convince me about the way that they quantify mitochondrial morphology and distinguish NB lineages/cells. The mitochondrial images presented in Figure 1B are clearer in the revised manuscript, but I doubt whether they are sufficient to allow accurate quantifications of mitochondrial morphology. On top of that, the quantifications were performed on regions manually chosen for those where mitochondria are most distinct, which does not necessary reflect the overall morphology in a given cell. I empathize with the authors as mitochondrial morphology quantification is challenging, but this is a very important part of the manuscript and the current data raise more doubts than adding any strength to the author’s conclusions.

**Response:**

We request the reviewer to note that change in mitochondrial morphology in the same mutants and for the same genes has been carried out in other tissues. It is already known that loss of Opa1 and Marf leads to mitochondrial fragmentation and Drp1 loss causes mitochondrial fusion. We aimed to assess mitochondrial morphology in the type II neuroblasts and the mINPs that we are performing the knockdowns. There are 8 such cells in each brain lobe. Electron microscopy would be ideal, but we do not have these facilities on campus, and it has been impossible to travel to perform these experiments elsewhere. Other tissues are used routinely for assessing the change in mitochondrial morphology. Therefore, we used fluorescence microscopy to probe the change in mitochondrial morphology in type II neuroblast lineages. STED has a better resolution as compared to confocal microscopy.

We have classified mitochondrial morphology into clustered, fragmented and intermediate and obtained percentage phenotype in the type II neuroblasts and mature INPs. We have added this data for all the genotypes in the revised manuscript.

We have carried out the mitochondrial size quantification from multiple sections from multiple cells across different brains. The reason for choosing a small region where mitochondria are discrete is to estimate an average mitochondrion size across different genotypes, which is not possible in the regions containing aggregated mitochondria. For Drp1SD, we quantify the regions close to the cluster, which have segregated mitochondria.

In the revised manuscript, we have also added mitochondrial morphology images in muscle cells in different genotypes in the supplementary figures to further document that the size of mitochondria is changed in these depletions. Our images are similar to those which have been documented in earlier studies.

Similarly, it is not entirely convincing that the authors were able to distinguish different neuroblast lineages as nearly all quantifications in Figure 2 rely on a single marker, except for Figure 2D for which representative images are unfortunately omitted. This deficiency, plus my concern in the mitochondrial quantification method, makes it hard to judge the reliability of the overall conclusion of the paper. I, therefore, cannot recommend this paper for publication at this stage.

Response:

We have performed co-staining of Dpn and Ase antibodies to identify mature INP (Dpn+) and GMC (Dpn-Ase+) populations in each type II lineage. Consistent with the previous observations, mature INPs were decreased in the Opa1 depletion, and this defect was suppressed by co-depletion of Drp1. Similarly, GMCs were lowered in Opa1 depletion, and this defect is also partially reversed on additional depletion of Drp1.

Reference

1.	[Deng Q, Guo T, Zhou X, Xi Y, Yang X, Ge W. Cross-Talk Between Mitochondrial Fusion and the Hippo Pathway in Controlling Cell Proliferation During Drosophila Development. Genetics. 2016;203: 1777–1788.](http://paperpile.com/b/bRdn0h/VrnW)

2.	[Wong KKL, Liao JZ, Shih CRY, Harden N, Verheyen EM. Hyperpolarized mitochondria accumulate in Hipk-overexpressing cells to drive tumor-like growth. J Cell Sci. 2020;133. doi:](http://paperpile.com/b/bRdn0h/mMyq)[10.1242/jcs.250944](http://dx.doi.org/10.1242/jcs.250944)

3.	[Deng H, Dodson MW, Huang H, Guo M. The Parkinson’s disease genes pink1 and parkin promote mitochondrial fission and/or inhibit fusion in Drosophila. Proc Natl Acad Sci U S A. 2008;105: 14503–14508.](http://paperpile.com/b/bRdn0h/qKsY)

4.	[Dorn GW 2nd, Clark CF, Eschenbacher WH, Kang M-Y, Engelhard JT, Warner SJ, et al. MARF and Opa1 control mitochondrial and cardiac function in Drosophila. Circ Res. 2011;108: 12–17.](http://paperpile.com/b/bRdn0h/qz66)

5.	[Trevisan T, Pendin D, Montagna A, Bova S, Ghelli AM, Daga A. Manipulation of Mitochondria Dynamics Reveals Separate Roles for Form and Function in Mitochondria Distribution. Cell Rep. 2018;23: 1742–1753.](http://paperpile.com/b/bRdn0h/tc38)

6.	[Tapia A, Palomino-Schätzlein M, Roca M, Lahoz A, Pineda-Lucena A, López Del Amo V, et al. Mild Muscle Mitochondrial Fusion Distress Extends Lifespan through an Early and Systemic Metabolome Reorganization. Int J Mol Sci. 2021;22. doi:](http://paperpile.com/b/bRdn0h/72uJ)[10.3390/ijms222212133](http://dx.doi.org/10.3390/ijms222212133)
