# Supplementary material for: Mitochondrial fusion regulates proliferation and differentiation in the type II neuroblast lineage in Drosophila
Source: PLoS Genet. 2022 Feb 14;18(2):e1010055. doi: 10.1371/journal.pgen.1010055 (PMC8880953; doi:10.1371/journal.pgen.1010055)
Supplement: Attachment — Submitted filename: PLOS Genetics Response to reviewers comments.docx [file pgen.1010055.s029.docx]

**We provide a point-wise response to the reviewers’ comments below:**

**Summary of changes made in response to the reviewer and editor comments:**

1. We have added stacked histogram plots to document the qualitative analysis of mitochondrial morphology into fragmented, intermediate and clustered in Figures 1, 3, 6 and S10.
2. We have changed the Marf RNAi mature INP image for mitochondrial morphology in Figure 1 for representing the fragmentation phenotype seen at the maximum percentage.
3. We have changed the description of neuron quantification in the type II neuroblast lineage as young neurons as they were quantified by staining for brightly expressing Prospero in the lineage. We made these changes in the introduction, results, figure legends and methods. We have added the label Neurons (bright Pros) on the Y axis in the graphs.
4. We have added the figures to PACE and uploaded the versions changed by the software for size and format in the revised submission.

**Reviewer #1:** I appreciate that the authors put a tremendous amount of effort into the revision. The manuscript has been improved significantly and most of my concerns have been addressed. Although I still have some reservation about the quantification of the area of mitochondria, the additional quantification of clustered/dispersed/fragmented mitochondria is helpful to some extent. I only have two suggestions.

**Response**: We thank the reviewer for working with us to improve the manuscript. We have documented the mitochondrial morphology in a qualitative manner from type II neuroblasts, mature INPs and muscle cells from different genotypes. Mitochondrial morphology in mitochondrial fusion and fission protein knockdown has been assessed previously for Opa1, Marf and Drp1 knockdown in different tissues and also with transmission electron microscopy. Since neuroblasts are small cells with abundant mitochondria and are present at deeper sections in the brain, we used STED super-resolution to get mitochondrial morphology area estimates from type II neuroblasts. Based on observations of mitochondrial distribution, we also divided mitochondrial distribution into fragmented, clustered and intermediate. We have added stacked histograms to show this distribution in the revised figures. Area estimates from STED sections are possible in regions where they are distinctly distributed. Hence we chose selected regions to estimate the area. The area estimate further attempts to support the descriptive analysis and highlight the difference between genotypes. We have therefore retained this in the revised manuscript.

First, If the authors insist on quantifying the number of neurons by labeling the type II NB lineages with mCD8-GFP driven by pntP1-GAL4 and Pros staining, they should state clearly that they are quantifying only young neurons, not the total number of neurons. Strong Pros expression is only in young/newly-born neurons, not in old neurons. Furthermore, the expression mCD8-GFP driven by pntP1-GAL4 certainly does not label all the cells in type II NB lineages (as they also stated in the response letter that sometimes they could not determine the boundary of the lineage based on the mCD8-GFP expression).

**Response**: As suggested, we have added the information of bright Pros positive cells being young neurons to the introduction, results, methods and legends section. We have determined the boundary of the lineage in *pnt* Gal4, UAS mCD8-GFP labeled lineages by staining with anti-GFP and using the brightness and contrast tool. We have added this information to the revised material and methods.

Second, I would suggest to provide bar graphs for the quantification of clustered/dispersed/fragmented mitochondria in the figures (1, 3, 6, S10) instead of providing the numerical data in the figure legends. Also, they should clearly describe these quantification data in the text of the Results part.

**Response**: As suggested, we have provided stacked histogram plots for the distribution of mitochondrial morphology into fragmented clustered and intermediate in Figures 1,3,6 and S10. We have also mentioned this quantification in the results section and provided the method in the materials and methods section.

**Reviewer #2:** The revisions were done to my satisfaction.

**Response**: We thank the reviewer for working with us to improve the manuscript.

**Reviewer #3:** Although the morphological changes of the mitochondrial network are better described in the revised version, the poor quality of microscope images in multiple figures still failed to convince us that they are sufficient to allow reliable quantifications of mitochondrial morphology. We, therefore, would like to keep our decision from the previous round of revision.

**Response:** We thank the reviewer for working with us to improve our manuscript. We have quantified the mitochondria by a qualitative analysis of describing them into fragmented, intermediate and clustered. This is carried out by examining cells across different depths in the Z stack acquired for the lineage. This is now represented as stacked histogram plots in Figure 1, 3, 6 and S10. The phenotype of mitochondrial clustering in Drp1 mutant is distinguishable with certainty and also remains in the combination of Drp1SD, opa1RNAi and Drp1SD, marf RNAi. The mitochondria appear as smaller dots in different optical planes in opa1 RNAi and marf RNAi. Since the type II neuroblasts and their progeny, such as mature INPs are small, and mitochondria are abundant, the qualitative analysis is an effective method. This method has been used in the previous analysis for describing mitochondria in various different cell types [[1]](https://paperpile.com/c/qtF1Xx/OGcl). We hope that this representation improves the documentation of changes in mitochondrial morphology.

As suggested, we have gone through all the figures showing mitochondrial morphology and replaced the marf RNAi mature INP image for mitochondrial morphology in Figure 1 for representing the phenotype of fragmentation observed at the highest percentage.

Reference:

1.	[Cao X, Wang H, Wang Z, Wang Q, Zhang S, Deng Y, et al. In vivo imaging reveals mitophagy independence in the maintenance of axonal mitochondria during normal aging. Aging Cell. 2017;16: 1180–1190.](http://paperpile.com/b/qtF1Xx/OGcl)
